# Degradation of Wheat Straw by *Streptomyces thermocarboxydus* XH2: Insights from Genomic and Transcriptomic Analyses

**DOI:** 10.3390/microorganisms14081798

**Published:** 2026-08-14

**Authors:** Tingyao Lv, Yushuo Zhang, Chao Wang, Qiuyang Jiang, Xiaotong Zeng, Feng Li, Dayong Xu

**Affiliations:** 1 Anhui Province Key Laboratory of Pollutant Sensitive Materials and Environmental Remediation, Huaibei Normal University, Huaibei 235000, China; 18362079282@163.com (T.L.); 15562580727@163.com (Y.Z.); 13225697527@163.com (C.W.); 18455770136@163.com (Q.J.); 13682967309@163.com (X.Z.); 2 School of Life Sciences, Huaibei Normal University, Huaibei 235000, China

**Keywords:** CAZy enzymes, genome sequencing, lignocellulose, straw degradation, *Streptomyces thermocarboxydus* XH2, transcriptome

## Abstract

Crop straw is an abundant lignocellulosic resource, but its efficient bioconversion is hindered by the recalcitrant structure of plant cell walls. This study integrated degradation phenotyping, enzyme activity assays, whole-genome analysis, and comparative transcriptomics to link the wheat-straw degradation performance of strain XH2 with its enzymatic and molecular responses. Strain XH2 was isolated from fully decomposed compost collected in Anhui Province, China, selected based on the formation of a distinct hydrolysis halo on CMC-Congo red agar, and deposited in the China Center for Type Culture Collection (CCTCC) under accession number CCTCC M 2025519. Morphological, cultural, phylogenetic, and genomic analyses identified strain XH2 as *Streptomyces thermocarboxydus*. Its degradation capacity was evaluated during 28 days of cultivation by measuring straw degradation, lignocellulosic components, scanning electron microscopy (SEM), and extracellular enzyme activities. *S. thermocarboxydus* XH2 caused marked disruption of the wheat-straw surface and achieved a degradation rate of 31.45%. Cellulose and hemicellulose contents decreased from 41.10% to 28.87% and from 30.72% to 16.85%, respectively, whereas lignin decreased from 8.28% to 6.30%. Endoglucanase activity, filter paper activity (FPase, an indicator of total cellulase activity), and xylanase activity peaked on day 7, reaching 35.99, 17.68, and 37.01 U/mL, respectively. Genome analysis revealed multiple genes encoding cellulases and hemicellulases. Comparative transcriptomic analysis after 72 h of cultivation in wheat-straw medium identified 1614 differentially expressed genes relative to Gause No. 1 medium, with major enrichment in ABC transporters and fructose and mannose metabolism. Most genes associated with polysaccharide degradation were upregulated. These findings link the degradation phenotype of *S. thermocarboxydus* XH2 to its enzymatic and molecular responses and support its further evaluation as a candidate for wheat-straw bioconversion under greenhouse and field conditions.

## 1. Introduction

With rapid socioeconomic development and population growth, environmental pollution and energy shortages have become increasingly serious concerns. As an agricultural powerhouse, China generates vast amounts of crop straw, a major byproduct of agricultural production and an important renewable lignocellulosic resource [1]. Preliminary statistics indicate that China produces approximately 900 million tons of crop straw annually, of which nearly 580 million tons are generated as agricultural processing byproducts [2]. However, the comprehensive utilization rate of these byproducts remains below 40% [3]. Without rational and efficient management, this not only results in substantial resource wastage but also contributes significantly to environmental pollution [4,5]. Therefore, promoting the bioconversion of crop straw biomass has become an urgent necessity [6,7].

Lignocellulose, the primary constituent of plant cell walls, is the most widely distributed and abundant biomass resource in nature [8,9]. Its structure is highly complex, comprising cellulose, hemicellulose, and lignin interconnected through extensive physical and chemical interactions [10]. Straw, a major lignocellulosic resource, holds great utilization potential [11], yet its practical development is hampered by unstable microbial performance, lignocellulose recalcitrance, and limited mechanistic understanding for optimization. Thus, identifying efficient lignocellulose-degrading microbes and elucidating their substrate deconstruction traits are critical for building more robust straw-treatment systems. Among lignocellulose-degrading microorganisms, *Streptomyces* species have attracted increasing attention because they are widespread in soil and plant-residue habitats and can secrete diverse carbohydrate-active enzymes involved in the degradation of cellulose, hemicellulose, and other plant polymers [12]. In addition, the filamentous growth pattern of *Streptomyces* facilitates substrate colonization and penetration, whereas their spore-forming ability and environmental adaptability support survival and activity under relatively unfavorable conditions [13,14]. These characteristics make *Streptomyces* promising candidates for straw biodegradation and bioconversion [15].

Despite these advantages, current studies on straw-degrading *Streptomyces* have mainly focused on strain screening, enzyme production, and overall biomass degradation performance. In contrast, relatively little is known about their substrate deconstruction preferences during straw degradation, particularly whether specific lignocellulosic components are preferentially attacked and how the corresponding enzymatic and metabolic pathways are regulated. This information is not only mechanistically important but also practically relevant, because component-level deconstruction patterns can help guide strain selection for specific pretreatment objectives, the design of complementary enzyme systems, and the optimization of cultivation conditions. Although *Streptomyces thermocarboxydus* has been reported as a thermotolerant *Streptomyces* species with potential enzyme-producing capacity, its role in wheat-straw degradation and its component-selective lignocellulose deconstruction strategy remain insufficiently characterized. Studies integrating degradation phenotype, enzyme activity, genome-based functional potential, and transcriptomic responses are still limited, especially for *S. thermocarboxydus* strains involved in straw bioconversion [16].

In this study, the taxonomic position of *S. thermocarboxydus* XH2 was determined through morphological, cultural, phylogenetic, and genomic analyses. Its ability to degrade wheat-straw lignocellulose was then evaluated by combining growth characteristics on lignocellulosic media, scanning electron microscopy (SEM), degradation rate and component measurements, and enzyme-activity analysis. Whole-genome sequencing and transcriptomic analyses were further integrated to characterize its functional potential and transcriptional responses during straw degradation [17]. The study aimed to determine whether *S. thermocarboxydus* XH2 exhibits component-selective deconstruction of wheat straw and to clarify the molecular basis of its lignocellulose degradation strategy. The novelty of this work lies in linking component-level degradation patterns with the induced carbohydrate-active enzymes and metabolic pathways of *S. thermocarboxydus* XH2, rather than evaluating the strain solely on the basis of overall degradation efficiency or individual enzyme activities. The findings provide a laboratory-scale mechanistic basis for optimizing XH2-related inoculum preparation, cultivation conditions, and enzyme combinations, and for designing subsequent greenhouse and field evaluations under more complex environmental conditions.

## 2. Materials and Methods

### 2.1. Materials and Reagents

#### 2.1.1. Strain

Strain XH2 was isolated from fully decomposed compost collected in Baishan Town, Suixi County, Huaibei City, Anhui Province, China. Briefly, the compost sample was suspended in sterile water, serially diluted tenfold, and spread onto Gause No. 1 medium for strain isolation. Single colonies were picked and purified by repeated streaking. The purified isolates were then inoculated onto sodium carboxymethyl cellulose (CMC)-Congo red plates to screen for cellulolytic activity. Strain XH2 produced a distinct hydrolysis halo on the CMC-Congo red plates, indicating cellulose-degrading ability, and was therefore selected for further investigation. This strain has been deposited in the China Center for Type Culture Collection (CCTCC) under accession number CCTCC M 2025519.

#### 2.1.2. Culture Media

The morphological characteristics of the strain were observed on Gause No. 1 medium [18], while its cultivation characteristics were evaluated on microcrystalline cellulose medium, CMC medium, xylan medium, lignin medium, wheat straw medium, soybean straw medium, corn stover medium, and corn cob medium [19]. All chemicals and reagents were of analytical grade and purchased from Sinopharm Chemical Reagent Co., Ltd. (Shanghai, China), unless otherwise specified.

Preparation of straw materials: Corn stover, corn cobs, wheat straw, and soybean straw were collected from rural areas surrounding Huaibei City, Anhui Province, China. The materials were thoroughly washed with deionized water to remove adhering soil and impurities, oven-dried at 80 °C to constant weight, cut into 2–5 cm pieces, ground using a plant grinder, and passed through a 20-mesh sieve. The resulting straw powder was stored in sterile sealed bags at room temperature until use. For medium preparation, the straw powder was directly added to the medium before autoclaving.

Gause No. 1 Medium: Soluble starch 20 g/L, KNO_3_ 1 g/L, K_2_HPO_4_ 0.5 g/L, MgSO_4_·7H_2_O 0.5 g/L, NaCl 0.5 g/L, FeSO_4_·7H_2_O 0.01 g/L, Agar 20 g/L, Distilled water 1000 mL, pH 7.2–7.4.

Microcrystalline Cellulose Medium: Microcrystalline cellulose 20 g/L, Urea 5 g/L, MgSO_4_·7H_2_O 0.5 g/L, (NH_4_)_2_SO_4_ 0.5 g/L, KH_2_PO_4_ 1 g/L, Agar 20 g/L, Distilled water 1000 mL.

CMC Medium: CMC 20 g/L, Urea 5 g/L, MgSO_4_·7H_2_O 0.5 g/L, (NH_4_)_2_SO_4_ 0.5 g/L, KH_2_PO_4_ 1 g/L, Agar 20 g/L, Distilled water 1000 mL.

Xylan Medium: Xylan (for signal pathway regulation, Sangon Biotech (Shanghai) Co., Ltd., Shanghai, China) 1 g/L, Urea 5 g/L, MgSO_4_·7H_2_O 0.5 g/L, (NH_4_)_2_SO_4_ 0.5 g/L, KH_2_PO_4_ 1 g/L, Agar 20 g/L, Distilled water 1000 mL.

Lignin Medium: Lignin (for signal pathway regulation, Sangon Biotech (Shanghai) Co., Ltd., Shanghai, China) 20 g/L, Urea 5 g/L, MgSO_4_·7H_2_O 0.5 g/L, (NH_4_)_2_SO_4_ 0.5 g/L, KH_2_PO_4_ 1 g/L, Agar 20 g/L, Distilled water 1000 mL.

Straw Medium: Using corn stover, corn cobs, wheat straw, and soybean straw as sole carbon sources respectively, added at 10 g/L ratio, with urea 5 g/L, MgSO_4_·7H_2_O 0.5 g/L, (NH_4_)_2_SO_4_ 0.5 g/L, KH_2_PO_4_ 1 g/L, Agar 20 g/L, and Distilled water 1000 mL.

CMC-Congo Red Medium: CMC 20 g/L, Urea 5 g/L, MgSO_4_·7H_2_O 0.5 g/L, (NH_4_)_2_SO_4_ 0.5 g/L, KH_2_PO_4_ 1 g/L, Congo red (indicator grade, dye content ≥85%, Sangon Biotech (Shanghai) Co., Ltd., Shanghai, China) 0.2 g/L, Agar 20 g/L, Distilled water 1000 mL [20].

Liquid Fermentation Medium: Wheat straw 20 g/L, Urea 5 g/L, MgSO_4_·7H_2_O 0.5 g/L, (NH_4_)_2_SO_4_ 0.5 g/L, KH_2_PO_4_ 1 g/L, Distilled water 1000 mL.

Wheat Straw Medium: Wheat straw 20 g/L, KNO_3_ 1 g/L, K_2_HPO_4_ 0.5 g/L, MgSO_4_·7H_2_O 0.5 g/L, NaCl 0.5 g/L, FeSO_4_·7H_2_O 0.01 g/L, Agar 20 g/L, Distilled water 1000 mL.

All media listed above were sterilized at 121 °C for 20 min.

### 2.2. Observation of Morphological Characteristics and Taxonomic Identification

Strain XH2 was identified using a polyphasic approach based on morphological, cultural, phylogenetic, and genome-based analyses.

Morphological and cultural characteristics were described with reference to the general taxonomic criteria for the genus *Streptomyces* in “Classification and Identification of *Streptomyces*” [21] and “Bergey’s Manual” [22]. Strain XH2 was streaked onto a variety of media, including microcrystalline cellulose, CMC, xylan, lignin, wheat straw, soybean straw, corn stover, and corn cob. An agar-solidified mineral medium without an added carbon source was included as a negative control. The composition of the control medium was identical to that of the basal mineral medium used for the substrate-containing plates, except that no carbon source was added. After incubation at 37 °C for 7 days, the characteristics of the strain were recorded, including colony morphology, the color of aerial and substrate hyphae, the production of soluble pigments, and growth performance on different media.

Additionally, the ultrastructure of the strain was examined using SEM (Regulus SU8220, Hitachi, Tokyo, Japan) after 7 days of culture at 37 °C on Gause No. 1 medium employing the insert method [23].

Molecular identification was carried out utilizing the 16S rDNA gene, which was extracted from the assembled whole-genome sequence. The sequences obtained were assembled and compared with reference strains in the EzBioCloud database (https://www.ezbiocloud.net/, accessed on 7 July 2025) [24] to establish phylogenetic relationships. A phylogenetic tree was subsequently established utilizing the Neighbor-Joining method in MEGA 7.0 software with 1000 bootstrap replications.

To further clarify the taxonomic position of strain XH2 at the species level, genome-based comparisons were performed using the whole-genome sequence. Details of genome sequencing are described below in Section 2.4. Average nucleotide identity (ANI) values were calculated using OrthoANI [25], while digital DNA–DNA hybridization (dDDH) values were estimated using the Genome-to-Genome Distance Calculator (GGDC 3.0) [26]. Species delineation was evaluated according to the generally accepted thresholds of 95–96% for ANI and 70% for dDDH.

### 2.3. Evaluation of Wheat Straw Degradation Ability

#### 2.3.1. Cellulose Hydrolysis and Growth on Lignocellulosic Media

The CMC–Congo red plate staining method was employed to visually evaluate the cellulose-degrading capacity of strain XH2 by exploiting the specific binding of Congo red to cellulose and the subsequent formation of hydrolysis rings.

Following the method described in Section 2.2, the ability of strain XH2 to grow on lignocellulosic substrates was evaluated on media containing microcrystalline cellulose, CMC, xylan, lignin, wheat straw, soybean straw, corn stover, and corn cob as the sole or major carbon source. The agar-solidified mineral medium without an added carbon source described in Section 2.2 was used as the negative control. Growth performance on each substrate was recorded after incubation at 37 °C for 7 days.

All phenotypic experiments were performed with at least three independent biological replicates.

#### 2.3.2. SEM Analysis

SEM was performed to investigate the morphological changes in wheat straw during biodegradation and to visualize microbial colonization and surface erosion caused by strain XH2.

A small amount of the activated culture was inoculated into 100 mL of CMC liquid medium (initial pH 7.0) in a 250 mL Erlenmeyer flask and incubated aerobically at 37 °C with shaking at 180 rpm for 7 days to prepare the seed culture. A 5% (*v*/*v*) inoculum from the 7-day-old seed culture was transferred into the liquid fermentation medium under the same culture conditions, and the time of inoculation was designated as 0 h. The cultures were maintained at 37 °C under aerobic conditions with shaking at 180 rpm throughout the experiment. All treatment groups were inoculated with the same 7-day-old seed culture prepared under identical conditions to minimize inoculum variation. Wheat straw samples were collected on days 1, 3, 7, 14, 21, and 28 post-inoculation for SEM observation [27].

The samples were fixed overnight at 4 °C in 2.5% glutaraldehyde dissolved in 0.1 M phosphate-buffered saline (pH 7.2). After fixation, the samples were washed three times with the same buffer for 15 min each, followed by dehydration through a graded ethanol series (10%, 30%, 50%, 70%, 90%, 95%, and 100%; 15 min per step). The dehydrated samples were then treated twice with isoamyl acetate for 20 min each to replace the ethanol. Subsequently, the samples were frozen at −80 °C for 12 h and freeze-dried for 12 h prior to SEM examination. For imaging, the dried samples were mounted onto aluminum stubs using double-sided carbon tape and sputter-coated with gold to enhance conductivity. Observations were performed using a field-emission SEM (Regulus SU8220, Hitachi, Tokyo, Japan) at an accelerating voltage of 3.0 kV. Images were acquired at various magnifications, and scale bars are included in each micrograph. Uninoculated wheat straw samples processed under the same conditions served as controls.

#### 2.3.3. Degradation and Composition Analysis of Wheat Straw

Wheat straw degradation experiments were performed as described in Section 2.3.2. Straw samples were collected on days 1, 3, 7, 14, 21, and 28 after inoculation for SEM observation and gravimetric analysis. Each treatment included three biological replicates, and three uninoculated controls were included in parallel to estimate abiotic mass loss during cultivation. After sampling, straw residues were rinsed three times with sterile water to remove residual medium and as much loosely attached bacterial biomass as possible, and then dried at 65 °C to constant weight before weighing. The apparent relative degradation rate was calculated as:

(1) $$\mathrm{RDR}\;=\;\left(W-W_{0}\right)/\;W\times 100\%,$$

*W* is the mass of wheat straw dried to constant weight prior to fermentation (g); *W*_0_: Mass of wheat straw after treatment with the strain (g).

Note: Because trace microbial biomass may remain attached to the straw surface even after washing, the gravimetric values reported here represent apparent degradation rates.

The lignocellulosic composition of the straw was determined using the Van Soest sequential detergent method [28]. Briefly, dried straw samples were ground and subjected to sequential extraction with neutral detergent and acid detergent solutions to determine neutral detergent fiber (NDF) and acid detergent fiber (ADF), respectively. The ADF residue was subsequently treated with 72% sulfuric acid to determine acid detergent lignin (ADL). After each extraction, the insoluble residue was filtered, washed, dried to a constant weight, and quantified gravimetrically. Hemicellulose content was calculated as the difference between NDF and ADF, cellulose content as the difference between ADF and ADL, and lignin content was represented by ADL.

#### 2.3.4. Enzyme Activity Assay

Following the cultivation method described in Section 2.3.2, fermentation broths were collected at various time points, centrifuged at 12,000 rpm for 5 min, and the supernatant was collected as crude enzyme solution for subsequent enzyme activity assays. All enzyme activity assays were performed in triplicate, and results are expressed as the mean ± standard deviation (SD).

Endoglucanase activity and total cellulase activity measured by the filter paper assay (FPase) were assayed with modifications to the method described by Ghose [29], whereas xylanase activity was assayed with modifications to the method of Bailey et al. [30]. The reducing sugars released during the enzymatic reactions were quantified using the 3,5-dinitrosalicylic acid (DNS) method described by Miller [31]. For endoglucanase, 0.1 mL of appropriately diluted crude enzyme was mixed with 2.0 mL of 1% (*w*/*v*) CMC-Na in 0.2 mol/L acetate buffer (HAc–NaAc, pH 4.8) and incubated at 50 °C for 20 min. For FPase, one strip of Whatman No. 1 filter paper (1 × 6 cm, approximately 50 mg) was incubated with 0.5 mL diluted crude enzyme and 2.0 mL of the same buffer at 50 °C for 60 min. For xylanase, 0.1 mL diluted crude enzyme was incubated with 0.9 mL of 1% (*w*/*v*) birch xylan at 37 °C for 30 min. After incubation, DNS reagent was added to terminate the reaction, followed by boiling for color development, cooling, dilution to 25 mL with deionized water, and absorbance measurement at 520 nm for endoglucanase and FPase and 540 nm for xylanase. Heat-inactivated crude enzyme (100 °C, 5 min) was used as the blank control. Glucose standard curves were used for endoglucanase and FPase, and a xylose standard curve was used for xylanase. Enzyme activities were calculated from the amount of reducing sugar released after blank correction and expressed as U/mL according to reaction time, enzyme volume, and dilution factor. One unit (U) was defined as the amount of enzyme releasing 1 μmol of reducing sugar or xylose per min under the assay conditions.

Laccase (Lac), lignin peroxidase (LiP), and manganese peroxidase (MnP) activities were determined using commercial assay kits (Suzhou Greese Biotechnology Co., Ltd., Suzhou, China) according to the manufacturer’s instructions under the specified reaction conditions. Lac activity was assayed with 2,2′-azinobis-(3-ethylbenzothiazoline-6-sulfonic acid) (ABTS) at 420 nm, LiP activity with resveratrol at 310 nm, and MnP activity with dimethoxyphenol (DMP) at 470 nm. Activities were calculated from blank-corrected absorbance changes using the formulas provided in the kits and expressed as U/mL; one unit (U) was defined as the amount of enzyme catalyzing the formation of 1 nmol of product per min.

Cellobiose dehydrogenase (CDH) activity was measured spectrophotometrically in 100 mM acetate buffer (pH 5.0) containing 1.0 mM cellobiose (analytical grade, Sangon Biotech, Shanghai, China), 200 μM 2,6-dichloroindophenol (DCIP; indicator grade, Sangon Biotech, Shanghai, China), and 20 μL crude enzyme at 37 °C. The decrease in absorbance at 520 nm was recorded over 6 min, and heat-inactivated enzyme was used as the blank control. CDH activity was calculated from the blank-corrected decrease in absorbance at 520 nm based on DCIP reduction and expressed as U/mL according to reaction time, enzyme volume, and dilution factor. One unit (U) was defined as the amount of enzyme reducing 1 μmol of DCIP per min under the assay conditions.

### 2.4. Whole-Genome Sequencing and Genome Annotation

A single colony of strain XH2 was inoculated into CMC liquid medium and incubated at 37 °C with shaking at 180 rpm for 7 days. The cells were then harvested by centrifugation at 6000 rpm for 10 min. Genomic DNA of strain XH2 was extracted using a genomic DNA extraction kit according to the manufacturer’s instructions (Sangon Biotech, Shanghai, China). After DNA quality assessment, whole genome sequencing was performed by Majorbio Bio-Pharm Technology Co., Ltd. (Shanghai, China) using a combination of PacBio RS II SMRT long-read sequencing and Illumina short-read sequencing.

Raw sequencing reads were quality-filtered using fastp (v0.20.0) to remove adapter sequences, low-quality reads, reads containing excessive ambiguous bases, and excessively short reads. The quality-filtered PacBio long reads and Illumina short reads were used for genome assembly and polishing. De novo genome assembly was performed using Flye (v2.9.2), and the resulting assembly was further polished with Illumina clean reads to improve base-level accuracy. The quality, completeness, and contamination level of the genome assembly were evaluated using CheckM (v1.2.2). When terminal overlaps were detected, the assembly was further inspected manually and validated by read mapping.

Protein-coding genes were predicted using GeneMarkS (v4.30). The predicted genes or protein sequences were functionally annotated by comparison against multiple databases, including NR [32], Swiss-Prot [33], Pfam [34], COG [35], GO [36], and KEGG [37].

### 2.5. Transcriptome Sequencing and Bioinformatic Analysis

#### 2.5.1. Cultivation of Strain XH2

Strain XH2 was inoculated simultaneously into Gause No. 1 medium and wheat straw medium and incubated at 37 °C with shaking at 180 rpm for 72 h. The culture broth was then centrifuged at 11,000 rpm for 10 min to collect bacterial cells. Samples obtained from Gause No. 1 liquid medium served as the control group and were designated as G1, G2, and G3, whereas samples obtained from wheat straw medium were designated as the experimental group and named J1, J2, and J3. Each sample was prepared in triplicate.

#### 2.5.2. Transcriptome RNA Extraction and Sequencing

The collected bacterial cells were thoroughly washed 3 times with sterile PBS buffer, rapidly frozen in liquid nitrogen for 10 min, and subsequently stored at −80 °C. RNA extraction was carried out following the protocol provided with the RNA extraction kit (Sangon Biotech, Shanghai, China). Samples that met quality standards were then sent to Majorbio Bio-Pharm Technology Co., Ltd. (Shanghai, China) for cDNA library construction and Illumina sequencing.

Raw reads were quality-filtered using fastp to remove adapters, low-quality reads, and the resulting clean reads were used for downstream analysis. The complete genome sequence of strain XH2 assembled in this study was used as the reference genome. Clean reads were aligned to the reference genome of strain XH2 using Bowtie2. The reference genome was aligned against databases including NR, Swiss-Prot, Pfam, COG, GO, and KEGG to comprehensively acquire gene functional information and analyze the annotation status across these databases. Gene expression levels were quantified using RSEM (v1.3.3) and normalized to fragments per kilobase of transcript per million mapped reads (FPKM). The resulting expression matrix was subsequently used for Venn diagram analysis, correlation analysis, and principal component analysis (PCA). DEGs between the two groups were identified using DESeq2 (v1.42.0), with a significance threshold of adjusted *p*-value < 0.05, |log_2_(fold change)| ≥ 1 and FPKM ≥ 1. GO enrichment analysis of DEGs was performed using GOATOOLS (v1.4.4), and CAZyme annotation was conducted using dbCAN (v3.0.7). All bioinformatics analyses were performed on the Majorbio Cloud Platform (https://cloud.majorbio.com, accessed on 13 May 2025).

### 2.6. Quantitative PCR Analysis

#### 2.6.1. RNA Extraction and cDNA Synthesis

Total RNA was extracted according to the manufacturer’s instructions using TRIzol reagent (Takara, Beijing, China). Briefly, 1 g wet cell mass was ground to powder in liquid nitrogen, followed by the addition of 1 mL TRIzol reagent for complete lysis. After chloroform extraction, isopropanol precipitation, and washing with 75% ethanol, the RNA pellet was dissolved in 50 μL DEPC-treated water. RNA concentration and purity were determined using a NanoDrop 2000 spectrophotometer (Thermo Scientific, Waltham, MA, USA). The A260/A280 ratios ranged from 1.8 to 2.1, indicating that the RNA quality was suitable for subsequent experiments.

For reverse transcription, the entire RNA preparation (approximately 50 μL) was used as template, and cDNA was synthesized using the PrimeScript™ FAST RT reagent Kit with gDNA Eraser (Takara, Beijing, China) in a total reaction volume of 60 μL. The reverse transcription program was 37 °C for 15 min, followed by 85 °C for 5 s to inactivate the reverse transcriptase. The resulting cDNA samples were subjected to 5-fold serial dilution with five dilution levels (1, 1/5, 1/25, 1/125, and 1/625).

#### 2.6.2. RT-qPCR Amplification

RT-qPCR was performed on a LightCycler^®^ 96 Real-Time PCR System (Roche, Basel, Switzerland) using TB Green^®^ Premix Ex Taq™ II FAST qPCR (Takara, Beijing, China). Each 25 μL reaction mixture contained 12.5 μL 2× TB Green Premix Ex Taq II Fast qPCR, 1 μL each of forward and reverse primers (final concentration 0.4 μmol/L), 2 μL cDNA template, and ddH_2_O to a final volume of 25 μL. The amplification program consisted of an initial denaturation at 95 °C for 30 s, followed by 40 cycles of 95 °C for 5 s, 60 °C for 30 s, and 72 °C for 30 s. A melting curve analysis was then performed from 65 °C to 95 °C, with fluorescence acquisition every 0.5 °C, to confirm amplification specificity. Each sample was analyzed with three technical replicates. The functional annotations of the genes evaluated by RT-qPCR, together with primer sequences, amplicon lengths, amplification efficiencies, and Ct values, are provided in Supplementary File S1: Table S1.

The amplification efficiencies of the target genes and the reference gene were calculated from the slope of the standard curve using the formula:

(2) $$E=\left(10^{-1/slope}-1\right)\times 100\%$$

#### 2.6.3. Reference Gene Selection and Normalization

To identify a suitable internal reference gene, six candidate reference genes, including *16S rRNA*, *frr*, *rplA*, *thyA*, *gyrB*, and *rpoA*, were evaluated based on their RT-qPCR performance. Ct values were used to assess transcript abundance, with lower Ct values indicating higher expression levels. The average Ct values of these candidate genes ranged from 12 to 24. Among them, *16S rRNA* showed the lowest mean Ct value (12.52), whereas *gyrB* showed the highest mean Ct value (23.79). The overall expression ranking was: *16S rRNA* > *rpoA* > *thyA* > *frr* > *rplA* > *gyrB*.

**Figure 1:**
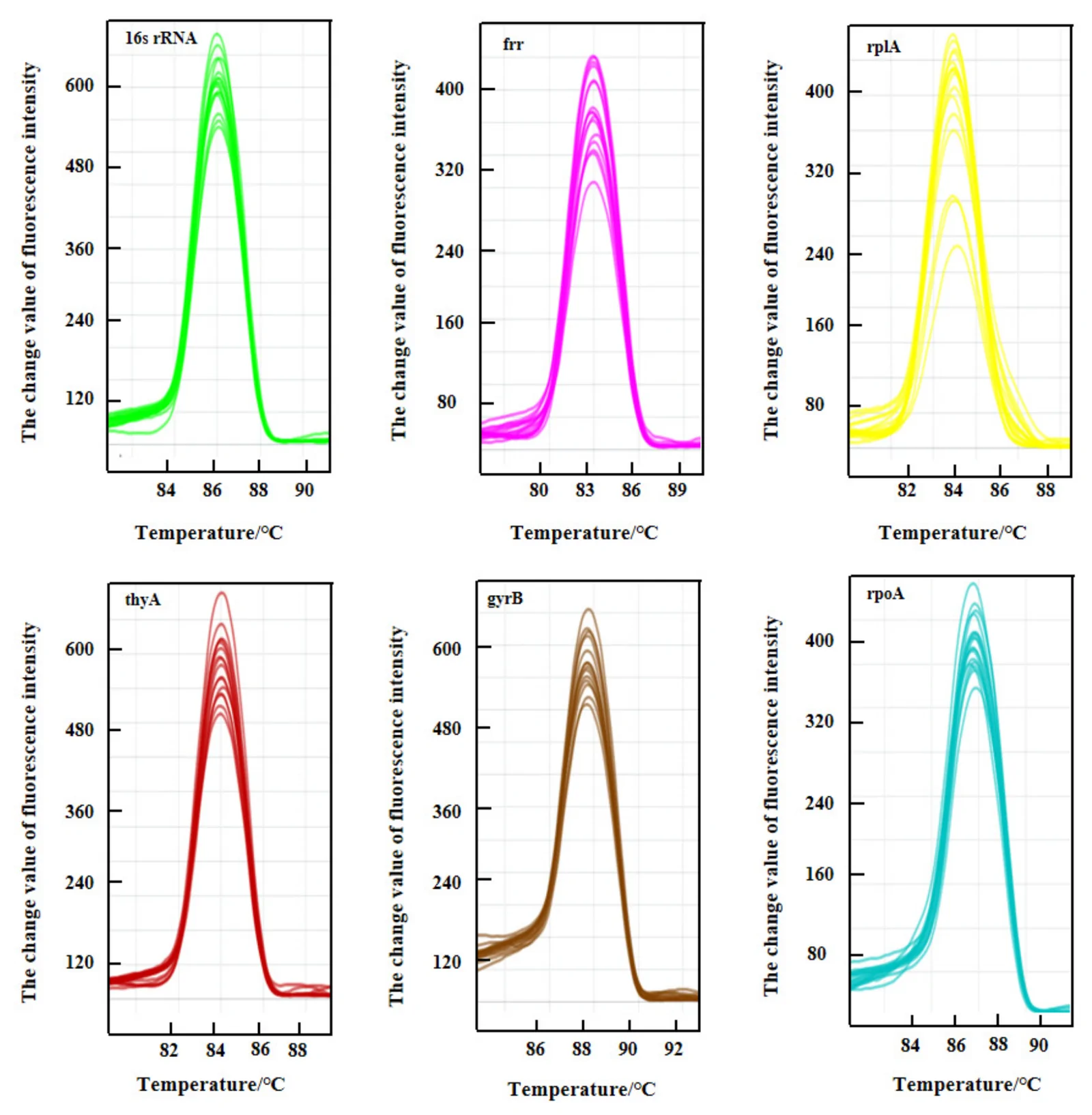
Melting curves of candidate reference genes.

The specificity of primer amplification was further confirmed by melting curve analysis (Figure 1). All six candidate reference genes showed clear single peaks between 80 and 90 °C, with no evidence of primer-dimer formation or nonspecific amplification. Based on its stable amplification performance and expression level, *16S rRNA* was selected as the internal reference gene.

For RT-qPCR data analysis, the expression levels of target genes were normalized using *16S rRNA* as the internal reference gene to minimize technical variation caused by differences in RNA extraction, reverse transcription efficiency, cDNA input, and amplification conditions. Relative expression levels were calculated using the 2^−ΔΔCt^ method [38].

### 2.7. Statistical Analysis

Data are presented as the mean ± SD of three independent experiments. For each independent experiment, three technical replicates were averaged to obtain one value used for statistical analysis. Differences among groups were analyzed using one-way analysis of variance (ANOVA), followed by Tukey’s honestly significant difference (HSD) test for multiple comparisons. A *p* value < 0.05 was considered statistically significant. All statistical analyses were performed using SPSS 26.0.

## 3. Results

### 3.1. Morphological, Cultural, and Taxonomic Identification of Strain XH2

**Figure 2:**
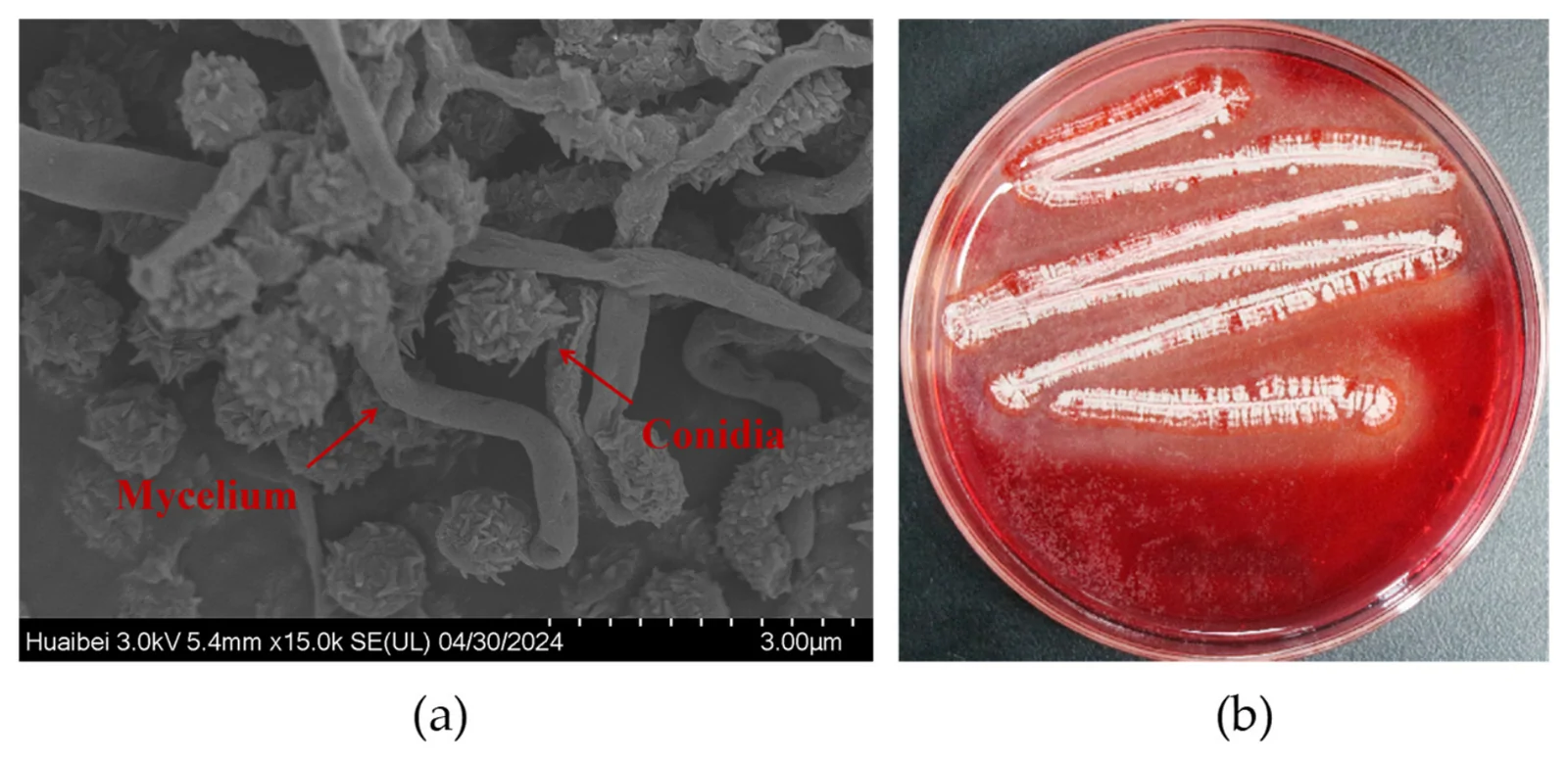
Micro-morphology and cellulolytic activity of strain XH2. (**a**) SEM observation of the strain; (**b**) Growth of the strain on CMC-Congo red plate.

Strain XH2 exhibited typical morphological characteristics of members of the genus *Streptomyces*. Morphological Characteristics: SEM (Figure 2a) revealed that the mycelium of strain XH2 exhibits a filamentous-cylindrical morphology, and its spores possess spiny projections on the surface.

**Figure 3:**
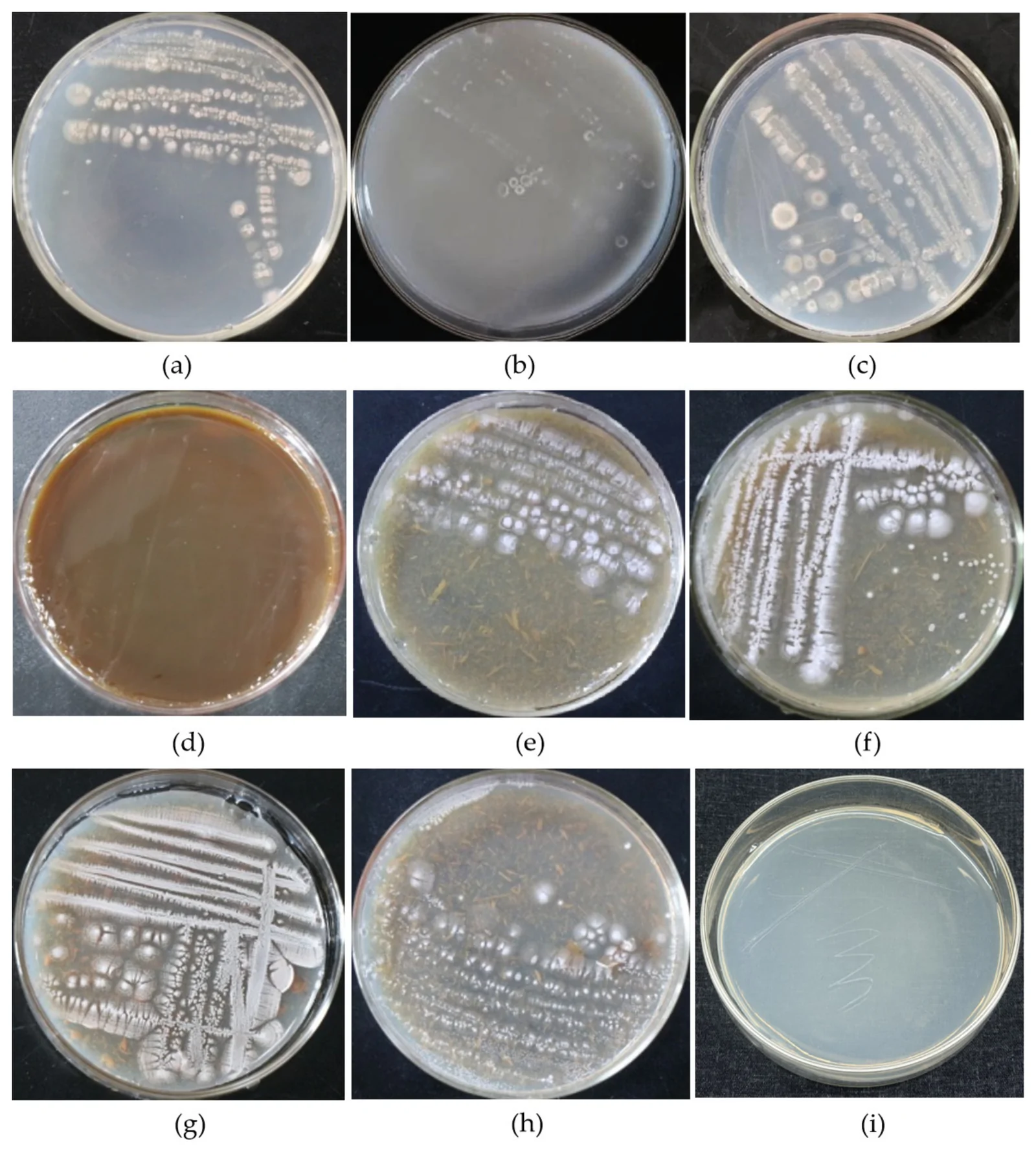
Morphological characteristics of strain XH2 on different media. (**a**) Microcrystalline cellulose medium; (**b**) CMC medium; (**c**) Xylan medium; (**d**) Lignin medium; (**e**) Wheat straw medium; (**f**) Soybean straw medium; (**g**) Corn stover medium; (**h**) Corn cob medium; and (**i**) agar-solidified mineral medium without an added carbon source.

The strain showed distinct growth characteristics on different media (Figure 3). The colonies of strain XH2 exhibit a wrinkled surface morphology. They appear white after 2 days of incubation and gradually turn black over approximately 7 days. The colonies are villous, firm, and dry in texture, with a dense structure that adheres tightly to the agar surface and is difficult to peel off. The strain produces melanin, which darkens both the colonies and the surrounding culture medium. These morphological and cultural characteristics were consistent with those commonly observed in the genus *Streptomyces*.

**Figure 4:**
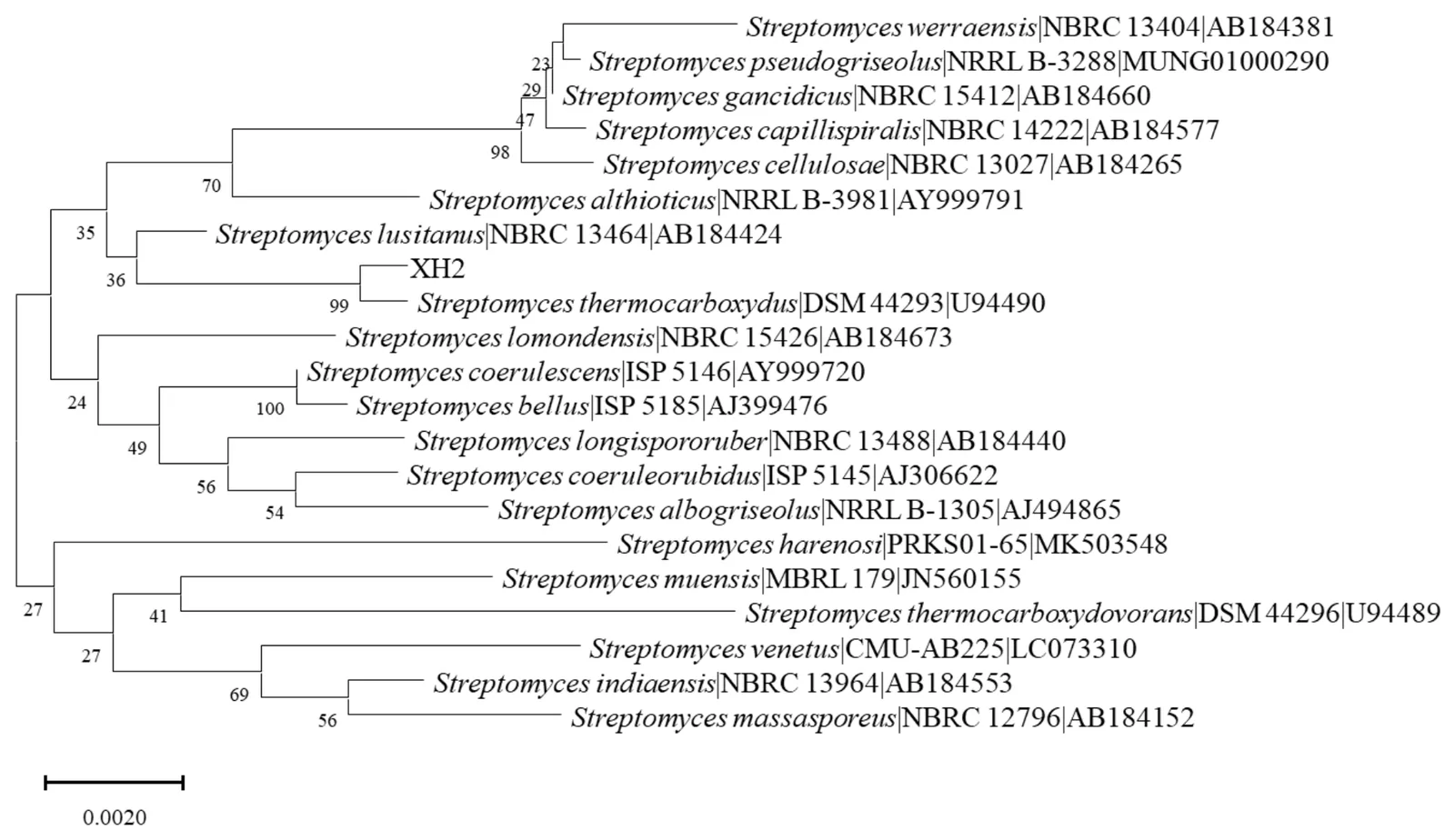
Phylogenetic tree of strain XH2 and similar strains based on 16S rDNA.

To further clarify the taxonomic position of strain XH2, its 16S rDNA sequence was compared with sequences in the EzBioCloud database to assess its phylogenetic relationships. A phylogenetic tree was constructed using the Neighbor-Joining method in MEGA 7.0. As shown in Figure 4, strain XH2 belonged to the genus *Streptomyces* and clustered most closely with *S. thermocarboxydus* DSM 44293, with which it shared the highest 16S rDNA sequence similarity.

The whole-genome sequence of strain XH2 was compared with that of *S. thermocarboxydus* JCM 10368, the type-strain equivalent of *S. thermocarboxydus* DSM 44293. The OrthoANIu and dDDH values were 99.27% and 94.10%, respectively.

Taken together, the morphological and cultural characteristics, 16S rDNA gene phylogeny, and genome-based taxonomic analyses collectively and consistently support the identification of strain XH2 as *Streptomyces thermocarboxydus*. Therefore, the isolate was designated as *S. thermocarboxydus* XH2 throughout this study.

### 3.2. The Degradation Potential of S. thermocarboxydus XH2 on Wheat Straw

#### 3.2.1. Growth Characteristics on Lignocellulosic Substrates

Distinct hydrolysis zones were observed around colonies of *S. thermocarboxydus* XH2 on CMC–Congo red plates after 7 days of incubation (Figure 2b).

*S. thermocarboxydus* XH2 was able to grow on several lignocellulosic substrates, although marked differences in growth were observed among media; a comparison of growth is shown in Table 1. Specifically, *S. thermocarboxydus* XH2 exhibited good growth on CMC, microcrystalline cellulose, xylan, and various straw-based media, with particularly vigorous growth on corn cob and corn stover media (Figure 3). It also grew stably and formed distinct colonies when wheat straw was used as the sole carbon source. In contrast, only negligible growth was observed on the lignin medium, and no evident growth was detected on the agar-solidified mineral medium without an added carbon source.

#### 3.2.2. SEM Observations of Wheat Straw During Biodegradation

**Figure 5:**
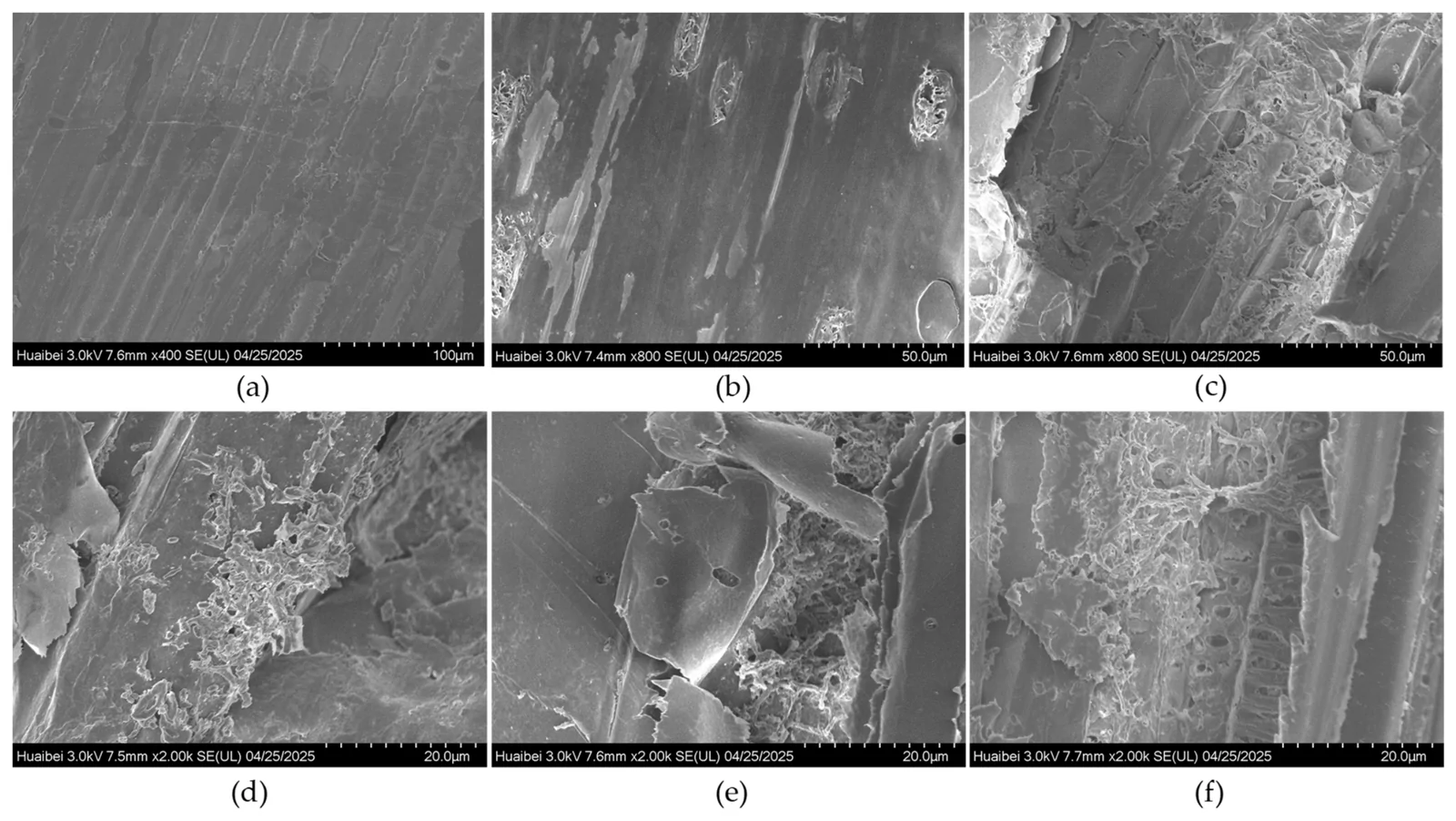
Representative SEM images showing the surface morphology of wheat straw in the control and XH2-treated groups during biodegradation. (**a**) Control, ×400 (scale bar = 100 μm); (**b**) day 1 and (**c**) day 7, ×800 (scale bar = 50 μm); (**d**) day 14, (**e**) day 21, and (**f**) day 28, ×2000 (scale bar = 20 μm). Note: Different magnifications were selected to better visualize the progressive structural changes occurring at different stages of biodegradation. All images were acquired under identical SEM operating conditions, and representative images from each treatment are shown.

Figure 5 shows representative SEM images of wheat straw from the control and XH2-treated groups during biodegradation. The control samples maintained an intact, compact, and relatively smooth surface throughout the incubation period, with no apparent structural damage. In contrast, wheat straw treated with *S. thermocarboxydus* XH2 exhibited progressive and time-dependent morphological alterations. After 1 day of incubation, the straw surface became rough and uneven (Figure 5b). By day 7, localized surface disruption became more evident (Figure 5c). After 14 days, the outer surface was markedly degraded, with exposed fibrillar structures and visible microbial colonization (Figure 5d). As incubation progressed to days 21 and 28, the straw surface exhibited extensive collapse, accompanied by numerous cracks, cavities, and fragmented structures (Figure 5e,f).

#### 3.2.3. Degradation of Wheat Straw by *S. thermocarboxydus* XH2

**Figure 6:**
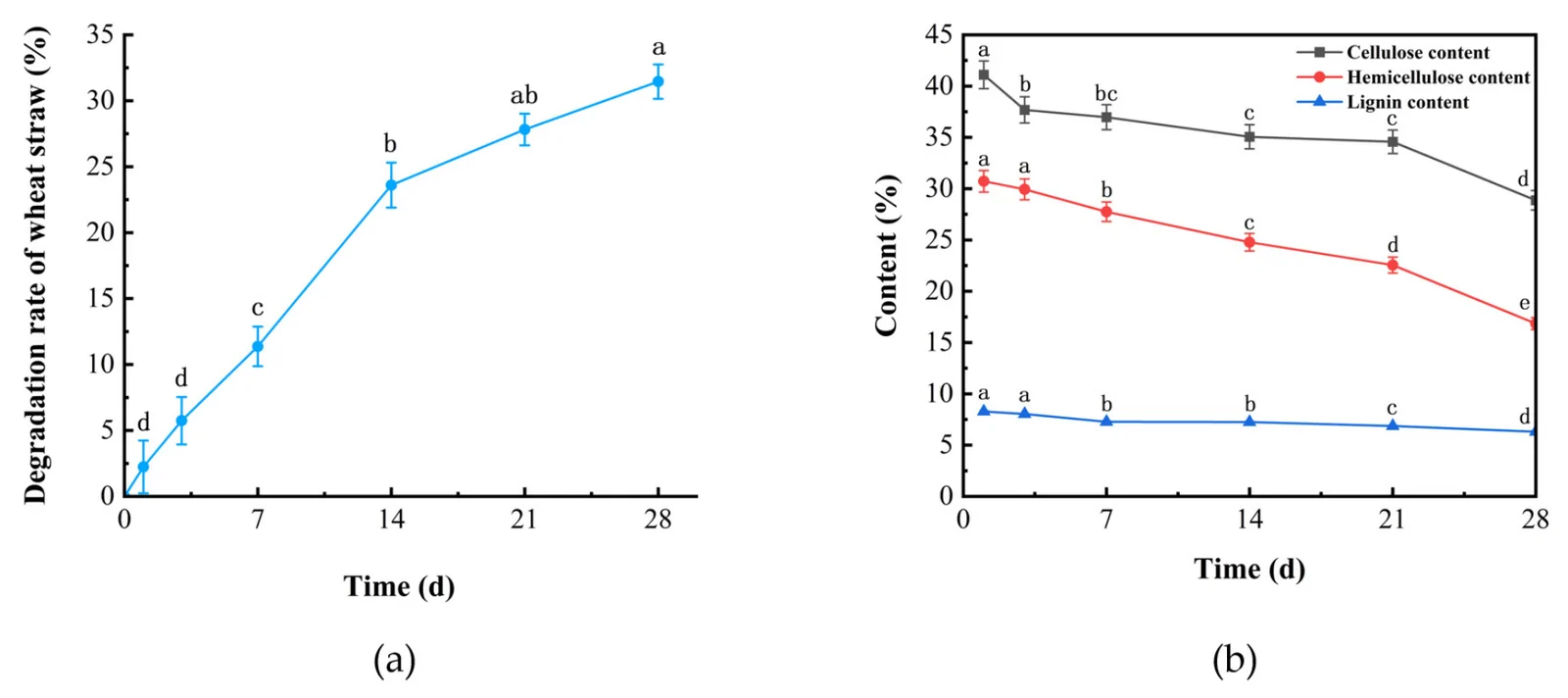
Degradation of wheat straw by *S. thermocarboxydus* XH2. (**a**) Straw degradation rate; (**b**) Content of cellulose, hemicellulose, and lignin. Note: Data are presented as mean ± SD from three biological replicates (*p* ≤ 0.05). The letters represent salience and the error bars represent SD.

This study further measured the mass loss and changes in the content of major components during the cultivation process (Figure 6) to quantitatively evaluate the degradation capacity of *S. thermocarboxydus* XH2.

As shown in Figure 6a, the dry weight of the straw gradually decreased as the incubation period increased. After 28 days of incubation, the mass loss rate of the straw in the *S. thermocarboxydus* XH2 treatment group reached 31.45%.

Analysis of the residue composition (Figure 6b) revealed significant differences in the changes in various components of the straw. Compared with the initial straw, the cellulose and hemicellulose contents were significantly reduced after treatment with *S. thermocarboxydus* XH2. After 28 days of cultivation, the cellulose content decreased from the initial 41.10% to 28.87%, and the hemicellulose content decreased from 30.72% to 16.85%. In contrast, the lignin content showed a much smaller decrease, from 8.28% to 6.30% over the entire cultivation period.

#### 3.2.4. Dynamic Changes in Lignocellulolytic Enzyme Activity

**Figure 7:**
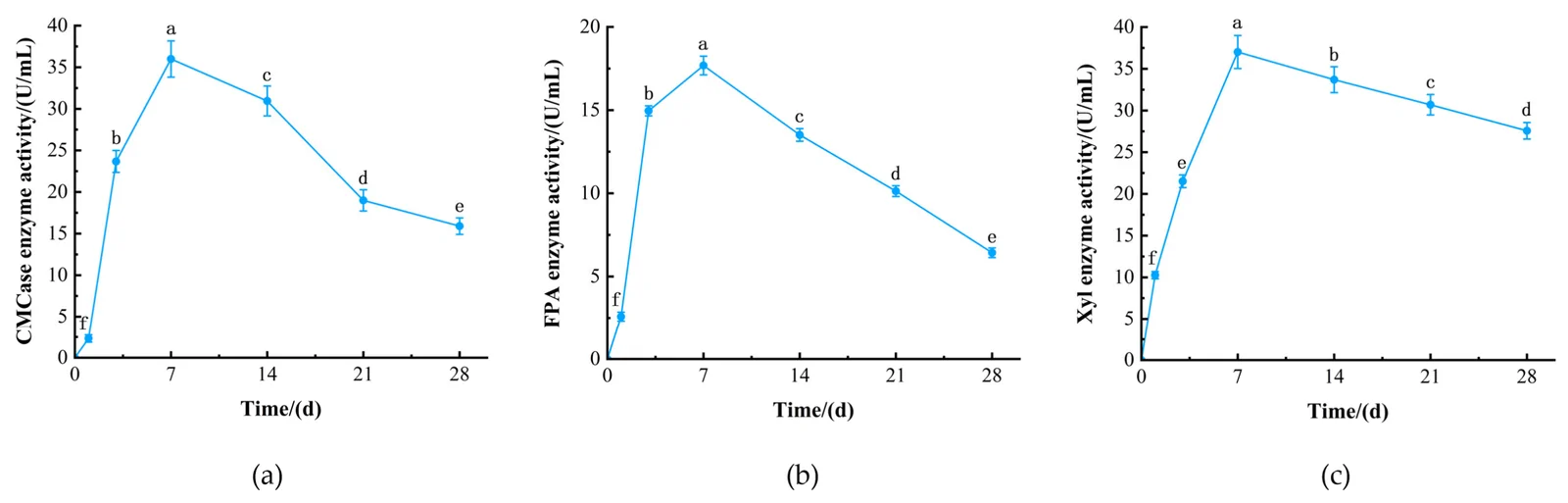
Changes in lignocellulose-degrading enzyme activities during fermentation. (**a**) CMCase activity; (**b**) FPase activity; (**c**) Xylase activity. Note: Data are presented as mean ± SD from three biological replicates (*p* ≤ 0.05). The letters represent salience and the error bars represent SD.

To further investigate the degradation of wheat straw by *S. thermocarboxydus* XH2, we measured changes in the activity of extracellular cellulases, xylanases, and lignin-degrading enzymes during cultivation. The results are shown in Figure 7.

At 3 days of fermentation, the CMC and FPase enzyme activities of *S. thermocarboxydus* XH2 were 23.67 U/mL and 14.96 U/mL, respectively (Figure 7a,b). As fermentation continued, enzyme activities gradually increased, peaking on day 7, at which point the activities were 35.99 U/mL and 17.68 U/mL, respectively. Ultimately, after 28 days of enzyme production fermentation, the CMC enzyme activity of *S. thermocarboxydus* XH2 decreased to 15.9 U/mL, and the FPase enzyme activity decreased to 6.43 U/mL. Xylanase activity followed a similar trend to that of CMC and FPase enzymes (Figure 7c), with overall enzyme activity showing an initial increase followed by a decrease. At day 3 of fermentation, the xylanase activity of *S. thermocarboxydus* XH2 was 33.7 U/mL. At 7 days of fermentation, enzyme activity increased significantly, reaching a peak of 37.01 U/mL. As fermentation progressed, enzyme activity decreased rapidly; after the 28-day enzyme production fermentation was completed, the xylanase activity of *S. thermocarboxydus* XH2 decreased to 27.57 U/mL.

To clarify the role of the *S. thermocarboxydus* XH2 in the degradation of lignin components during wheat straw decomposition, this study measured the activities of lignin-related enzymes, including Lac, LiP, MnP, and CDH. The results (Supplementary File S1: Table S2) showed that the activities of Lac, LiP, and MnP initially increased and subsequently declined during cultivation, displaying a temporal pattern similar to those of CMCase, FPase, and xylanase. However, their measured activities remained extremely low throughout the fermentation period and approached the detection limit at several sampling points. CDH activity was also generally low, with only a brief increase observed during the middle of the culture period.

### 3.3. Genomic Characteristics of S. thermocarboxydus XH2

To elucidate functional genes of *S. thermocarboxydus* XH2, whole-genome sequencing was performed. The genome was successfully assembled into a complete genome consisting of a single chromosome, and no plasmid was detected. A total of 178,615 reads were obtained, corresponding to an average sequencing depth of 180.88×. The genome of *S. thermocarboxydus* XH2 was 7,282,199 bp in size and had a GC content of 72.22% (Table 2). Genome quality assessment showed a completeness of 100% and a contamination level of 0%. Based on the complete sequence, a CGView genome map (Supplementary File S1: Figure S1) and a Circos plot (Supplementary File S1: Figure S2) were generated. GeneMarkS software predicted a total of 6572 coding genes, spanning 6,391,722 bp with an average length of 972.57 bp and a GC content of 72.51%, resulting in a gene-to-genome ratio of 87.77%. These coding genes were further annotated against multiple databases, including NR (6542 genes), Swiss-Prot (4340 genes), Pfam (5384 genes), COGs (5023 genes), GO (2810 genes), and KEGG (4400 genes).

**Figure 8:**
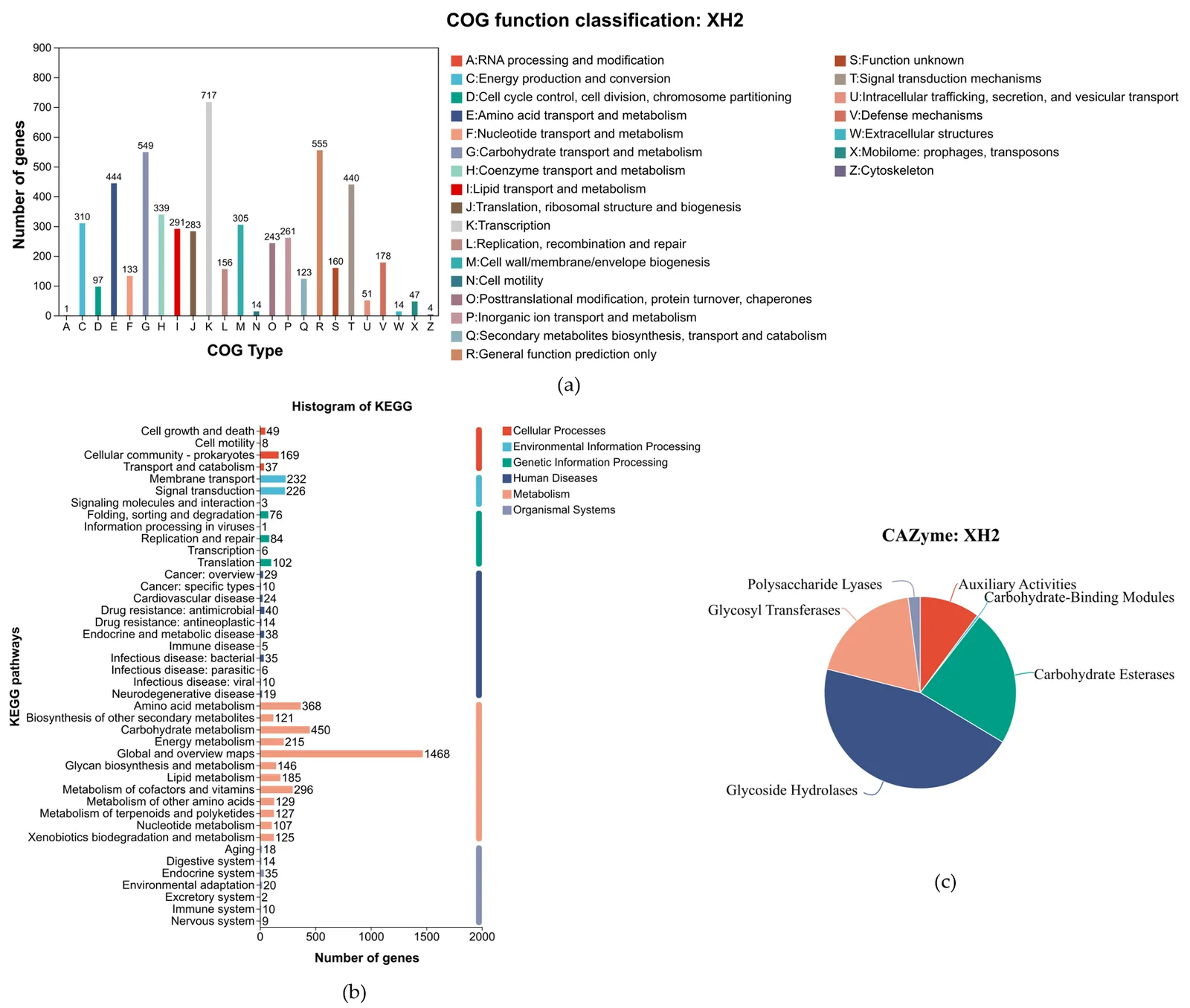
(**a**) COG functional classification statistics bar chart of *S. thermocarboxydus* XH2; (**b**) Pathway classification statistics bar chart of *S. thermocarboxydus* XH2; (**c**) Annotation Statistics Chart for Carbohydrate-Active Enzymes in *S. thermocarboxydus* XH2.

A total of 5023 functional genes were annotated based on the COG database (Figure 8a), accounting for 76.43% of all genes. Analysis revealed that genes involved in transcription were the most abundant, numbering 717, followed by 555 genes predicted to perform only general functions. Of these, 2450 genes were categorized into metabolic functions, including 549 genes directly associated with carbohydrate transport and metabolism.

Further functional annotation using the KEGG database identified 4400 genes associated with KEGG pathways (Figure 8b). Consistent with the COG annotation, metabolism remained the most extensively annotated category, encompassing 1564 genes. Within the Level 2 metabolic pathways, the Global and Overview Metabolism, Carbohydrate Metabolism, and Amino Acid Metabolism pathways contained the highest numbers of annotated genes, with 1468, 450, and 368 genes, respectively. The starch and sucrose metabolism pathway (ko00500) included 79 genes, among which numerous enzymes facilitating lignocellulose hydrolysis were identified, including endoglucanase (EC 3.2.1.4), hemicellulase (EC 3.2.1.91), and β-glucosidase (EC 3.2.1.21).

Additionally, all six CAZyme families—carbohydrate esterases (CEs), glycoside hydrolases (GHs), glycosyltransferases (GTs), polysaccharide lyases (PLs), carbohydrate-binding modules (CBMs), and enzymes with auxiliary activities (AAs) (Figure 8c)—were identified, with the GH family being the most abundant, accounting for approximately 45.3% of the total gene family, followed by the CE family at around 23%. These genes were further classified into 102 subfamilies, with CE4 and GT2_Glycos_transf_2 being the most prevalent, followed by GT4. Proteins encoded by *S. thermocarboxydus* XH2 genes (Table S3: Detailed table of CAZyme analysis) include endoglucanases (EC 3.2.1.4), primarily from the GH6, GH9, and GH12 families, and β-glucosidases (EC 3.2.1.21), mainly from the GH1, GH2, GH3, and GH16 families. Proteins encoded by xylanase (EC 3.2.1.8) genes predominantly belong to the GH10 and GH11 families, while those encoded by xylosidase (EC 3.2.1.37) genes mainly belong to the GH39 family. α-Arabinofuranosidase (EC 3.2.1.55) genes primarily encode proteins from the GH51 family. We found that the genome of *S. thermocarboxydus* XH2 contains 17 annotated cellulose-related genes, including genes from the GH1, GH6, and GH12 families; 59 annotated hemicellulose-related genes from the GH and CE families. Additionally, the genome of *S. thermocarboxydus* XH2 contains 10 annotated lignin-related genes, including genes from the AA1, AA2, and AA3 families.

### 3.4. Transcriptome Analysis

#### 3.4.1. Statistics and Quality Control of Illumina Sequencing Data

To identify genes involved in wheat straw degradation, whole-genome RNA-seq transcriptomic analysis was conducted. Following quality control of the raw transcriptome data, a total of 28.7 Gb of clean sequencing data was obtained, with each sample exceeding 4.23 Gb. As shown in Table 3, the base error rate was 0.01%, consistently below 3%. The Q20 base percentage exceeded 98.14% for all samples, while the Q30 percentage surpassed 92.15%. The average GC content was 72.22%, and the N50 average length was 12,059 bp.

#### 3.4.2. Gene Function Annotation and Functional Classification

A total of 8221 coding genes were annotated across six databases. The numbers of genes exhibiting significant sequence similarity to the GO, KEGG, COG, NR, Swiss-Prot, and PFAM databases were 1491 (18.14%), 3406 (41.43%), 6444 (78.38%), 8197 (99.71%), 5095 (61.98%), and 5586 (67.95%), respectively.

GO annotation of the transcriptome sequencing results (Supplementary File S1: Figure S3) revealed successful annotation of 1491 genes (18.14%). Among these, 1136 genes were involved in Biological Processes (BPs), 962 genes were associated with Cellular Components (CCs), and 2035 genes were related to Molecular Functions (MFs). Within each functional category, the most abundant entries were cellular process, cellular anatomical structure, and catalytic activity.

#### 3.4.3. Inter-Sample Expression Analysis

**Figure 9:**
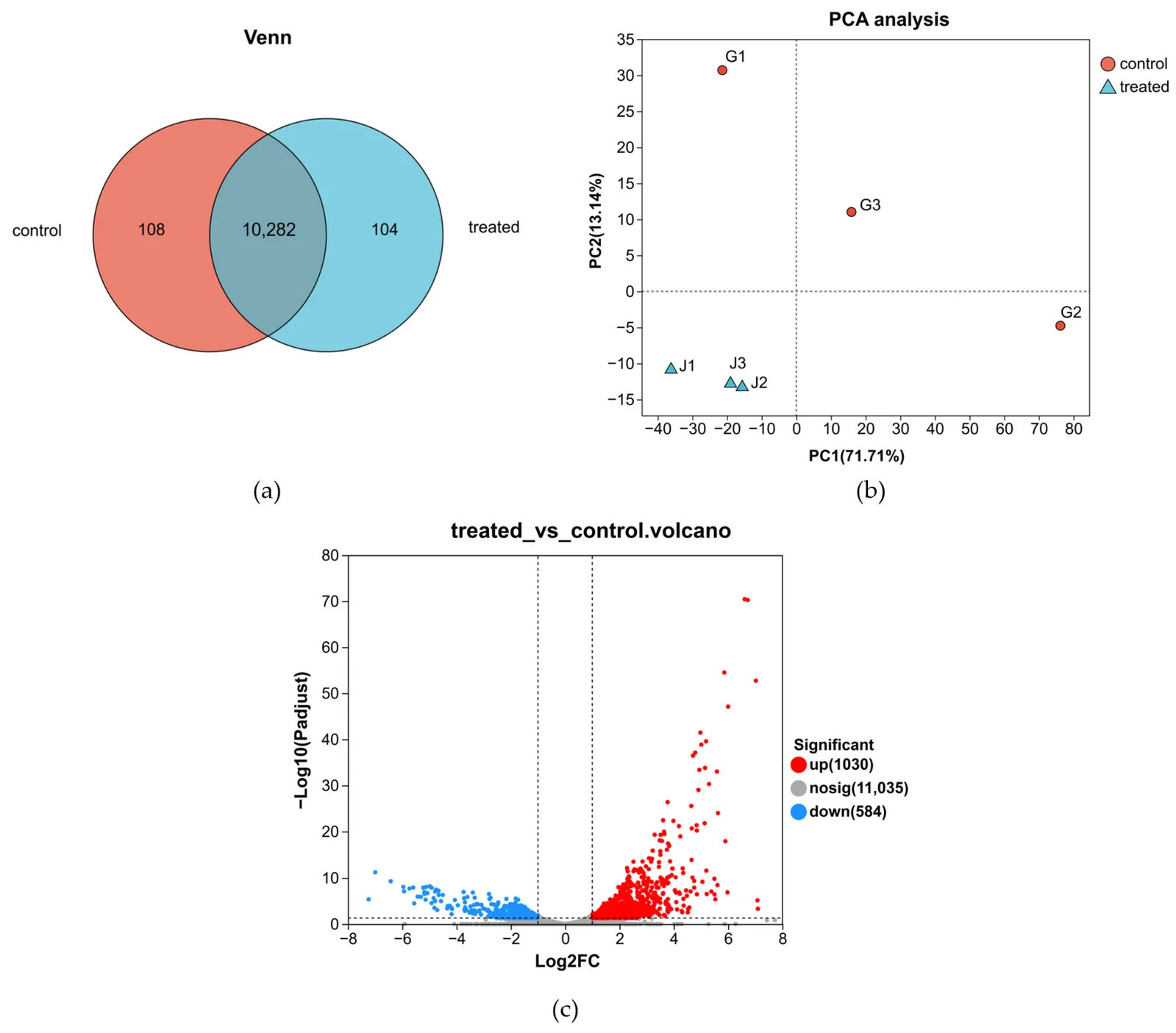
(**a**) Venn diagram of DEGs in *S. thermocarboxydus* XH2 cultured for 72 h on Gause No. 1 medium and wheat straw media; (**b**) PCA diagram of gene expression profiles based on six samples; (**c**) Volcano plot of DEGs in *S. thermocarboxydus* XH2 cultured for 72 h on Gause No. 1 medium and wheat straw medium.

To facilitate subsequent analysis of gene expression differences between the control and experimental groups, gene expression levels in both groups were first quantified (Figure 9a). The results showed that 10,282 genes were shared in the transcriptional profiles of both physiological states, reflecting high similarity in basal metabolism and cellular functions.

PCA based on expression levels demonstrated high reproducibility within each group. Transcriptomes of mycelia cultured in different media exhibited distinct patterns, with the control group (G1, G2, G3) and the experimental group (J1, J2, J3) clearly separated (Figure 9b). Samples from the same medium displayed minimal variation. These findings indicate minimal experimental error during the procedures and confirm that the detection and analysis model is suitable for subsequent data analyses.

#### 3.4.4. Differential Expression Analysis

To investigate DEGs during the growth of *S. thermocarboxydus* XH2 on Gause No. 1 medium and wheat straw medium, differential expression analysis was performed on samples cultured for 72 h. Genes were considered differentially expressed using the criteria of a adjusted *p*-value < 0.05, |log_2_(fold change)| ≥ 1 and FPKM ≥ 1, to rule out false positives that might result from DEGs with low expression levels. Volcano plots illustrating the DEGs across the growth stages of *S. thermocarboxydus* XH2 on both media are presented in Figure 9c. In total, 1614 DEGs were identified, including 1030 upregulated genes and 584 downregulated genes.

#### 3.4.5. GO Annotation and Functional Enrichment Analysis of DEGs

**Figure 10:**
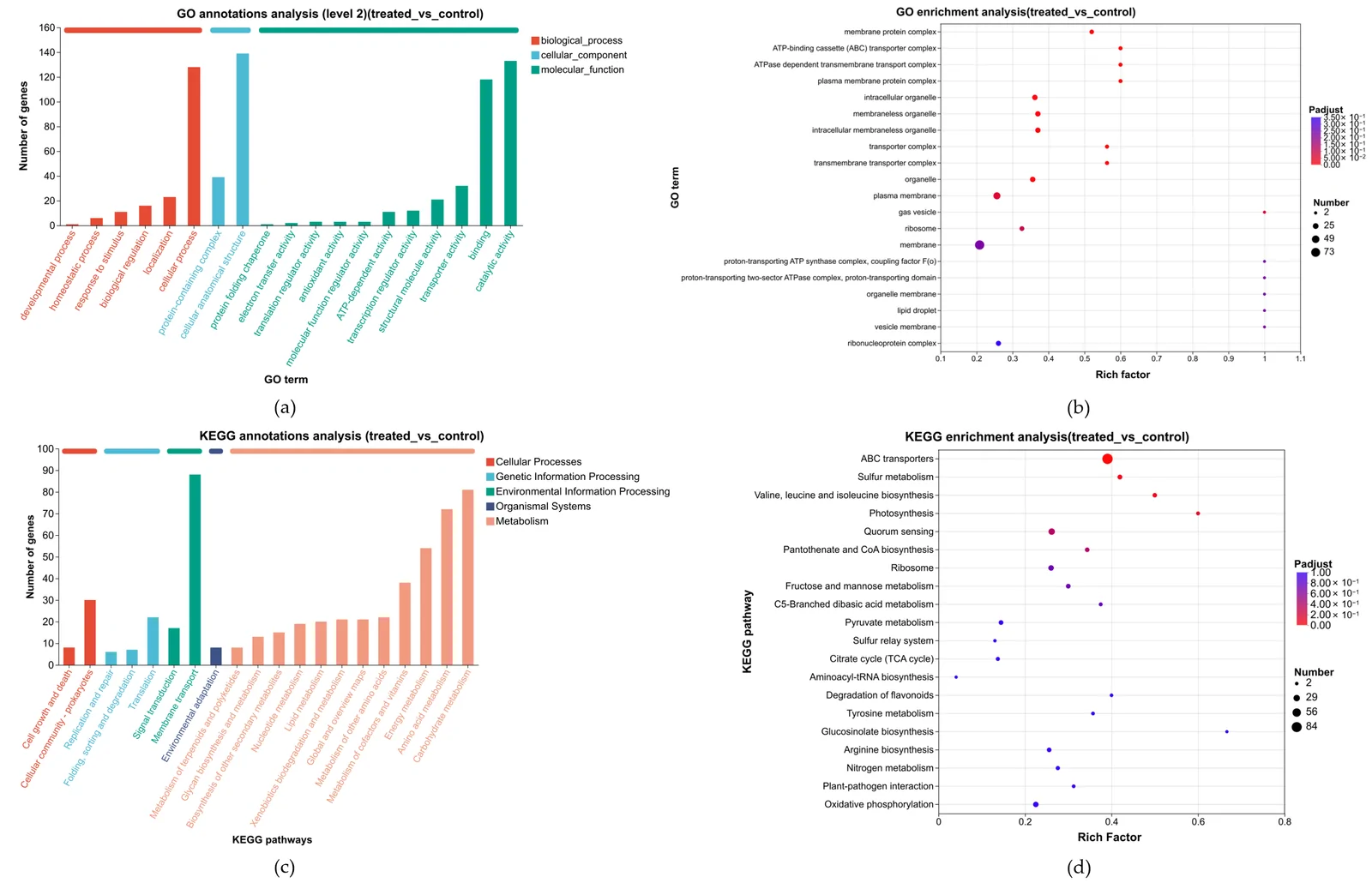
(**a**) Bar chart of GO classification statistics of DEGs of *S. thermocarboxydus* XH2 after 72 h of culture on Gause No. 1 medium and wheat straw medium; (**b**) Bubble chart of GO enrichment analysis; (**c**) Bar chart of pathway classification statistics of DEGs of *S. thermocarboxydus* XH2 after 72 h of culture on Gause No. 1 medium and wheat straw medium; (**d**) Bubble chart of KEGG enrichment analysis.

Annotation of the 1614 DEGs in the GO database is presented in Figure 10a, revealing 19 significantly altered GO terms. Within the MF category, 11 subcategories were identified, with catalytic activity and binding showing the highest numbers of DEGs, at 133 and 118, respectively, while the protein folding chaperone subcategory contained the fewest (1 gene). The CC category comprised two subcategories: cellular anatomical structure, with 139 DEGs, and protein-containing complex, with 39. Among the six subcategories within BP, cellular process contained the largest proportion of DEGs.

Further enrichment analysis of DEGs is presented in Figure 10b. The study revealed that DEGs were significantly enriched within the CC category, particularly in membrane protein complexes (GO:0098796) and ATP-binding cassette (ABC) transporter complexes (GO:0043190), encompassing 13 and 9 DEGs, respectively. The terms exhibiting the highest DEG counts were membrane (GO:0016020), plasma membrane (GO:0005886), and transporter activity (GO:0005215), with 73, 39, and 32 DEGs, respectively. Notably, genes related to non-membrane organelles (GO:0043228) and intracellular organelles (GO:0043229) were upregulated.

Subsequently, we analyzed the GO annotations of up- and downregulated DEGs. Within the MF category, the most strongly upregulated DEGs were involved in polysaccharide binding (GO:0030247) and cellulase activity (GO:0008810), while in the BP category, the highly upregulated DEGs participated in polysaccharide degradation processes (GO:0000272). In contrast, the most strongly downregulated DEGs were associated with cell adhesion (GO:0007155), metal ion transport (GO:0030001), and metal ion binding (GO:0046872).

#### 3.4.6. KEGG Enrichment Analysis of DEGs

KEGG annotation analysis (Figure 10c) revealed 20 significantly enriched pathways across five KEGG pathway categories. Among these, metabolic processes were predominant, encompassing four major pathways: carbohydrate metabolism (81 annotated genes), amino acid metabolism (72), energy metabolism (54), and cofactor and vitamin metabolism (38). Within the environmental information processing category, the membrane transport pathway was most prominent, comprising 88 annotated genes.

Using bioinformatics database resources, this study conducted KEGG pathway enrichment analysis on the DEGs and categorized them into various functional pathways (Figure 10d). A KEGG enrichment bubble plot was constructed based on the top pathways with the lowest *p*-values, with the vertical axis representing pathways and the horizontal axis indicating enrichment factors. This analysis identified 20 significantly enriched pathways, with ABC transporters and associated pathways showing particularly prominent enrichment. Upregulated genes were notably enriched in ABC transporters, valine, leucine, and isoleucine biosynthesis, photosynthesis, and sulfur metabolism pathways. Additionally, several other important pathways, including fructose and mannose metabolism, were highlighted. The significant enrichment of these pathways underscores their critical role in effectively regulating lignocellulose degradation.

#### 3.4.7. Screening and Expression Level Analysis of Potential Genes

Further analysis of the DEGs associated with straw degradation is presented in Table 4 and Supplementary File S1: Figure S4. Compared to growth on Gause No. 1 medium, after 72 h of cultivation on wheat straw medium, most genes encoding enzymes implicated in hemicellulose, cellulose, and lignin degradation were upregulated in *S. thermocarboxydus* XH2. Notable examples include endoglucanase (*gene6205*, *gene4909*), 1,4-β-d-glucanase (*gene5617*, *gene5618*), GH (*gene6205*, *gene6394*), polysaccharide monooxygenase (*gene6393*), β-glucosidase (*gene2389*), GH (*gene5120*), and hemicellulose-degrading enzymes such as 1,4-β-xylanase (*gene5907*, *gene5990*), xylanase (*gene2346*, *gene5915*), acetyl xylan esterases (*gene0624*, *gene5688*, *gene3430*, *gene5908*, *gene2537*), α-L-arabinofuranosidase (*gene5989*), and arabinogalactan β-galactosidase (*gene0551*). Among these, 1,4-β-d-glucanase, GH, and polysaccharide monooxygenase were significantly upregulated. Although the lignin-degrading gene (*gene4118*) was also upregulated, its expression was less pronounced and fewer in number compared to the genes for hemicellulose and cellulose. Furthermore, genes encoding FAD-dependent monooxygenases and MFS transporters were downregulated during straw degradation.

**Figure 11:**
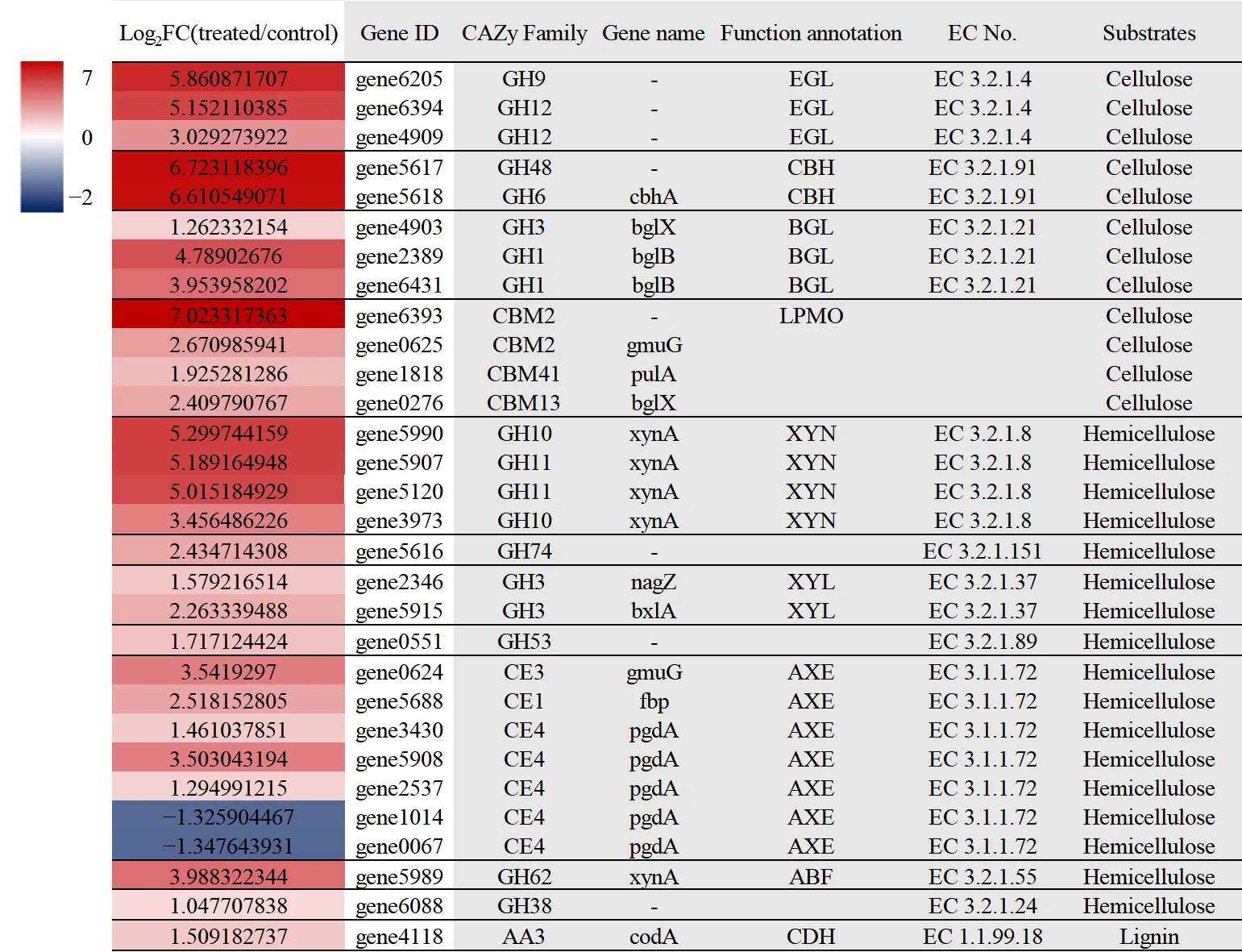
Expression of putative CAZyme DEGs associated with wheat straw lignocellulose degradation in the transcriptome. Note: Compared to the control transcriptome sample, dark red bars indicate significantly upregulated expression, while dark blue bars indicate significantly downregulated expression. 7, 0, −2 represent log_2_FC (treated/control) values; higher values indicate more pronounced upregulation.

To better understand the transcriptional regulation of genes encoding lignocellulolytic enzymes, we analyzed the differential expression of CAZy family genes during growth on wheat straw. A total of 57 CAZy DEGs associated with straw degradation were identified, with GHs (47.4%) as the dominant class, followed by GTs (21.0%), CEs (14.0%), CBMs (10.5%), AAs (5.3%), and PLs (1.8%). Among these 57 CAZy DEGs, 12 (40%), 17 (56.7%), and 1 (3.3%) were involved in cellulose, hemicellulose, and lignin degradation, respectively (Figure 11). These genes belong to diverse CAZy families with varying gene counts, with GHs remaining the predominant group. Cellulose-degrading genes included 1,4-β-d-glucan hydrolases (GH6, GH48), β-glucosidase (GH1), cellobiose-binding monomeric oxidase (CBM2), GHs (GH9, GH11, GH12, GH3, GH81, GH15, GH92, GT2, CBM13, CBM2), and GTs (GT4, GT2, GT83, CE4). Hemicellulose-degrading genes comprised xylanases (GH74, GH10, GH11), α-L-arabinofuranosidase (GH62), arabinogalactan-degrading enzymes (GH53), α-mannosidase (GH38), and N-acetylglucosamine-6-phosphate deacetylase (CE9). Lignin-degrading genes included cellobiose dehydrogenase.

### 3.5. RT-qPCR Validation

**Figure 12:**
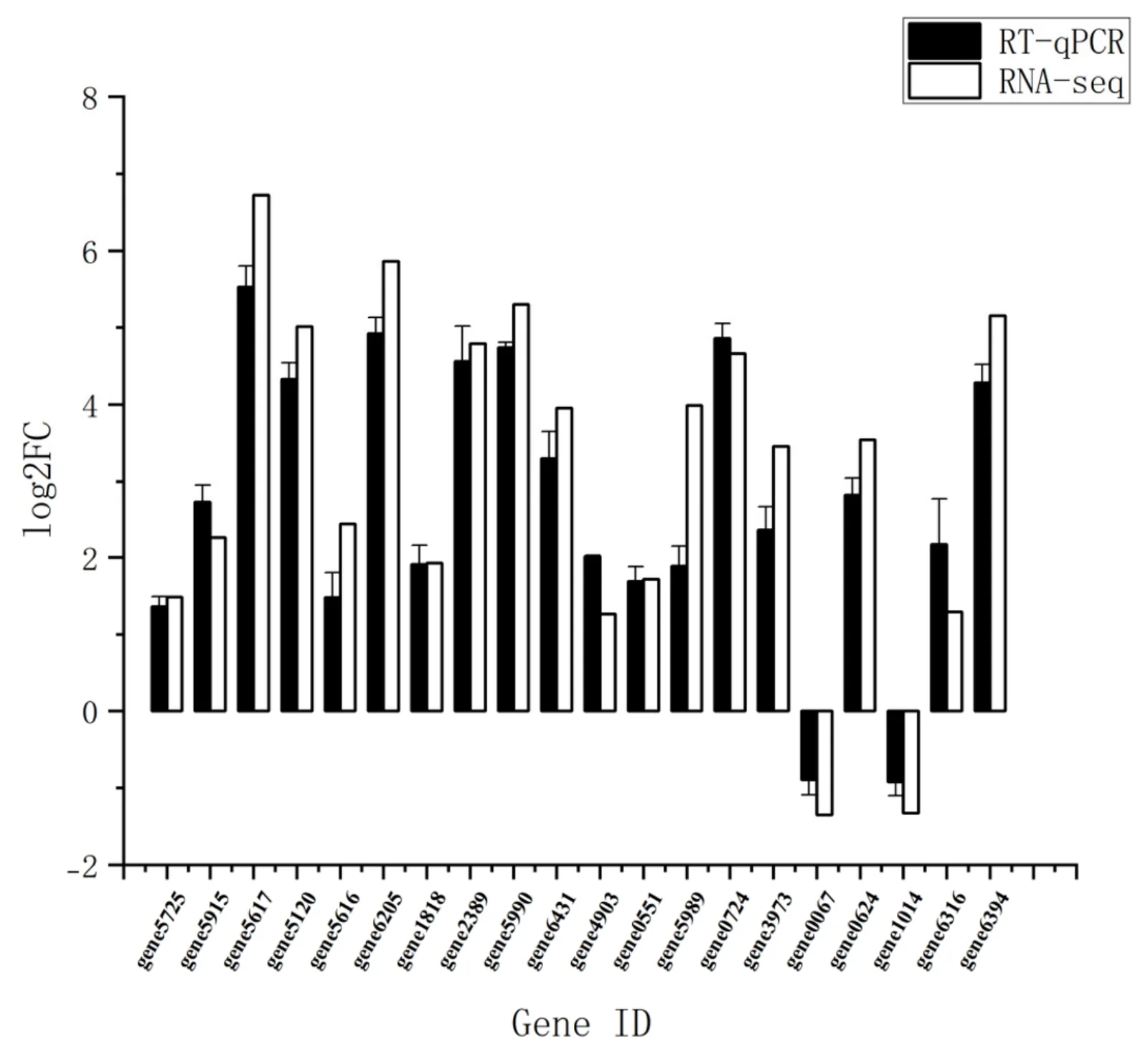
Validation of DEGs by RT-qPCR.

To validate the accuracy of the RNA-Seq data, the expression levels of 20 DEGs associated with lignocellulose degradation were examined using RT-qPCR. These genes mainly encoded enzymes or proteins related to lignocellulose degradation, including glycosyl hydrolases, β-glucosidases, xylanases, cellulase-related proteins, and carbohydrate-binding proteins. Detailed functional annotations of these genes are provided in Supplementary File S1: Table S1. The RT-qPCR results showed that 18 genes were upregulated and 2 genes were downregulated, which was consistent with the transcriptomic data (Figure 12).

## 4. Discussion

### 4.1. Taxonomic Identity and Substrate Utilization

Through a polyphasic approach integrating morphological, cultural, phylogenetic, and genomic analyses, strain XH2 was identified as *Streptomyces thermocarboxydus*. Compared with 16S rDNA analysis alone, the ANI and dDDH results provide stronger evidence for species-level assignment.

The combined growth, SEM, compositional, and enzyme activity results indicate that *S. thermocarboxydus* XH2 preferentially affected the carbohydrate fractions of wheat straw under the tested conditions. The strain grew on cellulose-, xylan-, and straw-based media but showed no visible growth on lignin medium. Consistently, cellulose and hemicellulose were altered more extensively than lignin during cultivation. These findings suggest that XH2 primarily uses cellulose- and hemicellulose-derived carbohydrates rather than lignin as carbon sources.

SEM revealed progressive surface roughening, fibril exposure, cracking, and structural collapse in XH2-treated straw. These changes may increase the accessibility of internal polysaccharides to extracellular enzymes. However, SEM provides morphological rather than chemical evidence and cannot independently identify the components removed from the straw. In addition, some surface damage may result from microbial attachment, physical fragmentation, or sample preparation. Therefore, the SEM observations should be interpreted together with the compositional and enzymatic results.

The early increase in CMCase, FPase, and xylanase activities suggests that polysaccharides in wheat straw induced the production of extracellular hydrolytic enzymes. The similar temporal patterns of these enzymes are consistent with a coordinated system for cellulose and hemicellulose deconstruction. Their later decline may have resulted from depletion of accessible substrates, feedback regulation by hydrolysis products, changes in culture conditions, or reduced extracellular enzyme stability, rather than complete cessation of gene expression. Although XH2 showed effective wheat straw conversion, direct comparison with previously reported strains should be made cautiously because degradation and enzyme activity measurements are strongly affected by substrate properties, culture conditions, and assay methods [39,40].

### 4.2. Genomic and Transcriptional Basis of Polysaccharide Utilization

XH2 contained a diverse repertoire of CAZymes, with GHs as the most abundant class, followed by CEs and GTs, whereas CBMs represented a relatively small proportion. GHs may directly hydrolyze cellulose, cellobiose, xylan, and hemicellulose side chains. CEs can remove substituent groups that restrict access to the hemicellulose backbone, while GTs are mainly involved in carbohydrate biosynthesis and modification. Although CBMs were less abundant, they may facilitate the binding of selected enzymes to insoluble polysaccharides. This repertoire provides a genetic basis for the broad substrate range of XH2. Nevertheless, genomic annotation reflects potential rather than confirmed activity, and differences in CAZyme numbers among microorganisms may also be influenced by genome quality and annotation methods [41,42].

Growth on wheat straw induced genes encoding putative cellobiosidases, endoglucanases, β-glucosidases, xylanases, accessory hemicellulases, and a lytic polysaccharide monooxygenase. These enzymes could act at different stages of polysaccharide conversion: endoglucanases cleave internal cellulose chains, cellobiosidases release shorter products from chain ends, and β-glucosidases convert cellobiose and cellodextrins into glucose [43,44]. Xylanases and accessory enzymes remove the xylan backbone and its side groups [45], whereas lytic polysaccharide monooxygenases may oxidatively disrupt recalcitrant polysaccharide surfaces [46]. Their coordinated induction is consistent with a multienzyme system targeting the heterogeneous carbohydrate matrix of wheat straw.

RT-qPCR confirmed the expression trends observed by RNA-seq. This agreement supports the reproducibility of the transcriptomic data but does not establish enzyme production or biochemical function. Translation, secretion, protein stability, and adsorption to insoluble straw may affect the relationship between transcript abundance and extracellular activity. The principal candidate genes therefore require validation through recombinant expression, enzymatic characterization, and genetic manipulation.

### 4.3. Coordination of Degradation, Transport, and Metabolism

GO and KEGG analyses showed enrichment of functions related to polysaccharide degradation, ABC transporters, and carbohydrate metabolism. Several oligosaccharide transport genes were also upregulated. Among them, cebE encodes a putative cellobiose-binding protein, cebG encodes an ABC transporter permease, and bxlE is associated with the binding or uptake of xylan-derived carbohydrates. Similar systems participate in cellobiose and cellotriose uptake in other *Streptomyces* species [47].

The co-upregulation of hydrolytic enzymes and carbohydrate transporters supports a provisional “degradation–transport–metabolism” model. In this model, extracellular enzymes release soluble sugars and oligosaccharides from cellulose and hemicellulose, ABC transport systems import these products, and intracellular carbohydrate pathways convert them into energy and metabolic precursors [48]. The enrichment of fructose, mannose, and galactose metabolism is also compatible with the intracellular utilization of straw-derived carbohydrates.

This model is based on gene annotations and expression patterns rather than direct transport measurements. ABC transporters are more likely to import soluble degradation products than to secrete extracellular degradative enzymes. Transport assays, isotope-labeled substrates, and targeted disruption of cebE, cebG, and bxlE are needed to determine their substrate specificity and physiological roles.

### 4.4. Limited Lignin-Degrading Capacity

Integration of genomic, transcriptomic, enzymatic, and compositional analyses indicates that XH2 has limited lignin-degrading capacity under the tested conditions. Although its genome encodes putative lignin-associated enzymes from the AA1, AA2, and AA3 families, only one AA3-family GMC oxidoreductase was upregulated. Genes encoding core lignin-modifying enzymes, including putative laccases and peroxidases, were not significantly induced.

AA3-family oxidoreductases may participate in electron transfer or H_2_O_2_ generation [48], but their activity alone is insufficient for extensive lignin depolymerization without the coordinated action of laccases, lignin peroxidases, manganese peroxidases, and redox mediators. Consistently, LiP, MnP, and Lac activities remained extremely low despite slight changes during fermentation. This suggests that an effective ligninolytic enzyme system was not established under the present conditions.

The observed decrease in lignin content should be interpreted cautiously because it was smaller than the changes in cellulose and hemicellulose. It may represent limited lignin modification or partial degradation rather than extensive depolymerization. Partial solubilization, physicochemical modification, analytical variation, and changes in the relative composition of the residue following preferential polysaccharide removal may also contribute to the apparent reduction.

The lignin-associated genes in the XH2 genome may be weakly induced or partially silent under the current culture conditions. Future studies could examine whether specific inducers, metal ions, redox mediators, improved oxygen supply, microbial consortia, or metabolic engineering can activate this oxidative machinery.

### 4.5. Limitations and Perspectives

This study was conducted under controlled laboratory conditions without greenhouse or field validation. Because the activity of XH2 may be affected by environmental conditions and indigenous microorganisms, future greenhouse and field trials should evaluate its colonization, straw-degrading performance, and effects on soil and plant growth.

Transcriptomic analysis was performed at a single time point and may not reflect the dynamic regulation of straw degradation. In addition, wheat straw medium and Gause No. 1 medium differed in both carbon source and overall nutrient composition; therefore, some DEGs may represent general nutritional responses rather than lignocellulose-specific regulation. Time-series analysis and defined media containing individual carbon sources would help distinguish these responses.

The inoculum was standardized by volume rather than biomass or viable-cell number. Residual microbial cells and particle loss during washing may also have affected the apparent mass loss. Future studies should standardize the initial biomass and determine absolute component mass balances, released sugars, and soluble aromatic products. Finally, the predicted functions of the principal CAZymes, transporters, and oxidative enzymes require direct biochemical and genetic validation.

Overall, XH2 appears to utilize wheat straw through coordinated extracellular polysaccharide hydrolysis, oligosaccharide transport, and intracellular metabolism, while its lignin-associated system remains weak. It therefore represents a potential candidate for cellulose- and hemicellulose-oriented bioconversion of agricultural residues.

## 5. Conclusions

*S. thermocarboxydus* XH2 showed a notable capacity to degrade wheat straw, particularly its cellulose and hemicellulose fractions. Morphological observation, SEM analysis, compositional determination, and enzyme activity assays consistently supported its lignocellulose-degrading ability. Genome annotation indicated that *S. thermocarboxydus* XH2 contains multiple genes encoding carbohydrate-active enzymes associated with straw deconstruction, while transcriptomic analysis suggested the induction of genes involved in polysaccharide degradation, transport, and carbohydrate metabolism during growth on wheat straw. These results suggest that *S. thermocarboxydus* XH2 has potential for wheat straw bioconversion and provide a basis for further investigation of its lignocellulose degradation mechanisms.

## Figures and Tables

**Table 1:** Comparison of the growth of *S. thermocarboxydus* XH2 on eight different culture media.

Strain	Culture Medium	Growth Score
*S. thermocarboxydus* XH2	Microcrystalline Cellulose Medium	+
	CMC Medium	++
	Xylan Medium	++
	Lignin Medium	−
	Wheat Straw Medium	++
	Soybean Straw Medium	++
	Corn Stover Medium	+++
	Corn Cob Medium	++++

Note: Growth was evaluated semi-quantitatively after incubation at 37 °C for 7 days. The growth score was assigned based on colony size, mycelial density, and surface coverage as follows: −, no visible growth; +, weak growth; ++, moderate growth; +++, good growth; and ++++, abundant growth.

**Table 2:** The basic genome features of *S. thermocarboxydus* XH2.

Features	Results	Features	Results	Features	Results
Total Reads No.	178,615	Gene Total Len (bp)	6,391,722	NR	6542
Total Bases (bp)	1,317,169,122	Gene Average Len(bp)	972.57	Swiss-Prot	4340
Largest (bp)	178,878	Gene Density (kb)	0.9	Pfam	5384
Average Len (bp)	7374.35	GC Content in Gene Region (%)	72.51	COG	5023
Reads N50(bp)	12,059	Gene/Genome (%)	87.77	GO	2810
Genome size (bp)	7,282,199	Intergenetic Region Len (bp)	890,477	KEGG	4400
GC content(%)	72.22	GC Content in Intergenetic Region (%)	70.15		
CDS No.	6572	Intergenetic Len/Genome (%)	12.23		

**Table 3:** Quality Control Data Statistics Table.

Sample Name	Raw Reads	Raw Bases (bp)	Raw Error Rate (%)	Raw Q20 (%)	Raw Q30 (%)	Clean Reads	Clean Bases (bp)	Clean Error Rate (%)	Clean Q20 (%)	Clean Q30 (%)
G1	30,257,140	4,568,828,140	0.0143	97.98	92.23	29,957,928	4,492,922,005	0.0137	98.4	92.96
G2	39,424,358	5,953,078,058	0.015	97.57	91.2	38,896,356	5,817,255,146	0.0142	98.14	92.15
G3	28,640,588	4,324,728,788	0.0128	98.58	94.55	28,376,362	4,234,039,324	0.0123	98.97	95.26
J1	32,029,658	4,836,478,358	0.0128	98.59	94.73	31,700,576	4,724,380,776	0.0122	99.01	95.5
J2	32,075,332	4,843,375,132	0.0127	98.62	94.74	31,785,188	4,719,764,649	0.0122	99.03	95.49
J3	32,105,032	4,847,859,832	0.0128	98.55	94.62	31,794,378	4,716,181,341	0.0123	99	95.44

**Table 4:** Differential expression and upregulation of the top 20 genes in the transcriptome.

Gene ID	Gene Name	Gene Description	Log_2_FC(Treated/Control)	Regulate	Family
*gene5618*	*cbhA*	cellulose 1,4-beta-cellobiosidase	6.610549071	up	GH6
*gene5617*	-	cellulose 1,4-beta-cellobiosidase	6.7231183956527	up	GH48
*gene6205*	-	glycosyl hydrolase family 5	5.8608717067251	up	GH9
*gene6393*	-	lytic polysaccharide monooxygenase	7.0233173626084	up	CBM2
*gene6428*	*cebE*	extracellular solute-binding protein	6.0018755485146	up	
*gene5912*	*bxlE*	extracellular solute-binding protein	4.9815410307086	up	
*gene5907*	*xynA*	1,4-beta-xylanase	5.1891649475677	up	GH11
*gene5120*	*xynA*	glycoside hydrolase family 11 protein	5.0151849289348	up	GH11
*gene2389*	*bglB*	beta-glucosidase	4.78902676	up	GH1
*gene6463*	-	hypothetical protein	4.7090514101785	up	
*gene6394*	-	glycosyl hydrolase family 5	5.1521103850959	up	GH12
*gene1396*	*actP*	cation acetate symporter	4.9438037439065	up	
*gene0252*	-	chitin-binding protein	5.589828282	up	AA10
*gene5990*	*xynA*	1,4-beta-xylanase	5.2997441587594	up	GH10
*gene1397*	-	DUF485 domain-containing protein	4.9091923450371	up	
*gene0367*	*katE*	Catalase	3.7748285477102	up	
*gene5554*	*ccrA*	crotonyl-CoA carboxylase/reductase	4.6433964171857	up	
*gene1563*	*terC*	TerC family protein	3.6069636082597	up	
*gene5989*	*xynA*	alpha-L-arabinofuranosidase	3.9883223439944	up	GH62
*gene2388*	*cebG*	carbohydrate ABC transporter permease	5.1366067398715	up	

## Data Availability

The 16S rRNA gene sequence has been deposited in GenBank under accession number PZ532785. The whole-genome sequencing data have been deposited in NCBI under BioProject accession number PRJNA1478474. The transcriptome sequencing data have been deposited in NCBI under BioProject accession number PRJNA1480174. Other data are included in the article/Supplementary Materials. Further inquiries can be directed to the corresponding author.

## Supplementary Materials

The following supporting information can be downloaded at: https://www.mdpi.com/article/10.3390/microorganisms14081798/s1, Supplementary File S1: Figure S1. Circular genome map of actinomycete XH2; Figure S2. Circos plot of actinomycete XH2; Figure S3. Statistical chart of GO annotation classification of transcriptomic sequencing results; Figure S4. Heat map analysis of the top 20 genes in the transcriptome; Table S1. Functional Annotation, Primer Information, and Reaction Parameters of Genes Evaluated by RT-qPCR; Table S2. Changes in enzyme activity associated with lignin degradation during the degradation process; Table S3: Detailed table of CAZyme analysis.

## Abbreviations

The following abbreviations are used in this manuscript:

AAO	aryl alcohol oxidase
ABC	ATP-binding cassette
ABF	α-L-arabinofuranosidase
ABTS	2,2′-azino-bis(3-ethylbenzothiazoline-6-sulfonic acid)
ADF	acid detergent fiber
ADL	acid detergent lignin
ANI	average nucleotide identity
AXE	acetyl xylan esterase
BGL	β-glucosidase
BP	biological process
CBH	cellobiohydrolase
CC	cellular component
CCTCC	China Center for Type Culture Collection
CDH	cellobiose dehydrogenase
CMC	carboxymethyl cellulose
DCIP	2,6-dichloroindophenol
dDDH	digital DNA–DNA hybridization
DEGs	differentially expressed genes
DMP	2,6-dimethoxyphenol
DNS	3,5-dinitrosalicylic acid
EGL	endoglucanase
FPKM	fragments per kilobase of transcript per million mapped reads
FPase	filter paper activity
Lac	laccase
LiP	lignin peroxidase
LPMO	lytic polysaccharide monooxygenase
MF	molecular function
MnP	manganese peroxidase
NDF	neutral detergent fiber
PCA	principal component analysis
SD	standard deviation
SEM	scanning electron microscopy
XYL	β-xylosidase
XYN	xylanase
