# Supplementary material for: Degradation of Wheat Straw by Streptomyces thermocarboxydus XH2: Insights from Genomic and Transcriptomic Analyses
Source: Microorganisms. 2026 Aug 14;14(8):1798. doi: 10.3390/microorganisms14081798 (PMC13515676; doi:10.3390/microorganisms14081798)
Supplement: Supplementary file 1 [file microorganisms-14-01798-s001.zip › Supplementary File 1.pdf]

Table S1. Functional Annotation, Primer Information, and Reaction Parameters of Genes Evaluated by RT-qPCR.

Gene	Gene description	Primer(5′-3′)	Product size (bp)	R^2	Slope	Efficiency(%)	Ct(±SD)
16s rRNA		CTTGGGCATCCTTGGTGATC<br>GTGTCTCAGTCCCAGTGTGG	143	0.998	-3.342	99.2	12.52(±0.07)
frr		ACATCATCCGGGTGGTGTTC<br>CAGAAGCTCATCCACCTGGG	239	0.989	-3.198	105.4	22.67(±0.08)
rplA		TCGAGTTCCGTGTCGACAAG<br>CTTGATGTAGCGGCCCTTG	149	0.993	-3.424	95.9	23.46(±0.14)
thyA		CCAAGGGCCTGATCAACTAC<br>GCATGAACTCGCGGAAGA	73	0.995	-3.193	105.6	20.39(±0.08)
gyrB		CTCACCCGTGAGGTTGTCGT<br>CGATGCAGTGGAACACCCAG	136	0.991	-3.362	98.4	23.79(±0.12)
rpoA		AAGAGGGTCGACGAATTCCG<br>CGGATGGACACACATGGACAC	115	0.993	-3.493	93.3	18.95(±0.19)
Gene5725	family 16 glycosylhydrolase	TGGAGAACACCCAAGGCATG<br>GTGTGGTAGGTGGTGTCGTC	214	0.990	-3.223	104.3	32.01(±0.13)
Gene5915	glycosyl hydrolase	TCCAACATCGATCCGACACC<br>GGTAGAGCTGGACCACCTC	178	0.982	-3.352	98.7	31.01(±0.18)
Gene5617	cellulose 1,4-beta-cellobiosidase	GTCGACGTTCCTCTCGATCC<br>CTATTCGAGAAGCTCCGCGT	166	0.993	-3.288	101.5	29.06(±0.28)
Gene5120	glycoside hydrolase family 11 protein	GTACGAGTCCGAGCAGAACC<br>AGAACGAGTAGTACATGCCGT	185	0.988	-3.283	101.7	30.12(±0.22)
Gene5616	xyloglucanase	GTTCGATTCCGACCGCATG<br>CATCATCGACGGCACCTTC	232	0.990	-3.280	101.8	32.19(±0.57)
Gene6205	glycosyl hydrolase family 5	CACAACGACATCACCCTCC<br>CTCGTGGTAGGTGTCGTACG	129	0.997	-3.338	99.2	29.72(±0.26)
Gene1818	pullulanase-type alpha-1,6-glucosidase	GGAAGATGGTCAAGGCGCTC<br>CTTGTACTCCTTGGCCCAGG	248	0.989	-3.199	105.4	31.72(±0.19)
Gene2389	beta-glucosidase	GCAACCAGATCTCCCTCAGCC<br>GTAGTAGTTGAGGCCCAGCG	251	0.986	-3.216	104.6	29.96(±0.32)
Gene5990	1,4-beta-xylanase	AGATGAAGATCGACGCGACC<br>GATGTGGTCGATCATCGCCT	191	0.991	-3.385	97.4	29.88(±0.21)
Gene6431	beta-glucosidase	CTCGGTCACCCTCAACATCC<br>GTGTCCTTGAGCAGGTCCTC	150	0.997	-3.309	100.5	30.96(±0.49)
Gene4903	carbohydrate-binding protein	ACTACTTCGACACGCACGAG<br>CGATGTCCTCCTCGGTCAAC	147	0.996	-3.361	98.4	31.83(±0.21)
Gene0551	arabinogalactan endo-1,4-beta-galactosidase	GCTGCTGATCGACTTCCAC<br>GTCCTTCTTGAGCTGCTCGT	103	0.993	-3.377	97.8	31.86(±0.53)
Gene5989	alpha-L-arabinofuranosidase	TGTCGCTGAAGGACTTCACC<br>GCACCCAGATGTTCTTCGG	207	0.994	-3.204	105.2	31.69(±0.51)
Gene0724	xylose isomerase	CGGTATGAAGGTCCCGATGG<br>GATGTTGCGGATGGTCTTGC	124	0.998	-3.335	99.4	29.81(±0.44)
Gene3973	endo-1,4-beta-xylanase	ACTACACGCTGCTGAAGGAC<br>GCATCCGCTGGTAGTACTCC	240	0.990	-3.163	107.1	31.52±0.39)
Gene0067	polysaccharide deacetylase	GTACGACTCCTCGCTCATGG<br>AGCGGTCCATCGTGAAGTAC	128	0.997	-3.319	100.1	32.53(±0.49)
Gene0624	cellulose-binding protein	ACACCGACATCGACTTCGTC<br>CGAAGTGCATGACAGCGATG	168	0.995	-3.394	97.1	29.95(±0.17)
Gene1014	bifunctional polysaccharide deacetylase/glycosyltransferase family 2 protein	AGATCGTCGTGATGCTGGAC<br>GAGGTTGAAGCCCATGACGT	176	0.995	-3.399	96.7	32.59(±0.27)
Gene6316	alpha/beta hydrolase	GGAACCTCGACAAGACCTGG<br>GACGGCAGGGTGTTGTAGAAG	118	0.997	-3.356	98.6	31.41(±0.36)
Gene6394	glycosyl hydrolase family 5	CGGCTGTCACTACACGAAC<br>CACCCGGTTGAACCAGATCA	199	0.993	-3.245	103.3	30.15(±0.44)

Table S2. Changes in enzyme activity associated with lignin degradation during the degradation process

Enzyme activity/ （U·ml^{-1}）	Time（d）					
	**1**	**3**	**7**	**14**	**21**	**28**
Lac	0.3704±0.05^{d}	0.4445±0.03^{cd}	0.7778±0.05^{a}	0.6028±0.04^{b}	0.5297±0.03^{bc}	0.3922±0.03^{d}
LiP	1.9359±0.07^{f}	4.3020±0.12^{b}	5.1624±0.09^{a}	3.9354±0.08^{c}	3.1178±0.08^{d}	2.3892±0.07^{e}
MnP	0.1821±0.01^{c}	0.2002±0.01^{bc}	0.2912±0.02^{a}	0.2357±0.02^{b}	0.2188±0.02^{bc}	0.1976±0.01^{bc}
CDH	0.0441±0.01^{d}	0.0833±0.01^{bc}	0.1397±0.01^{a}	0.1029±0.02^{b}	0.0634±0.01^{cd}	0.0518±0.01^{cd}

Note: Enzyme activities are expressed as U/mL. Activity units were defined according to the corresponding assay methods described in Section 2.3.4. The data represent the average of three measurements, with the numbers being the mean values ± standard deviation (SD) of the three replicates, and lowercase letters following the data in the same column indicate significant differences ($p < 0.05$).

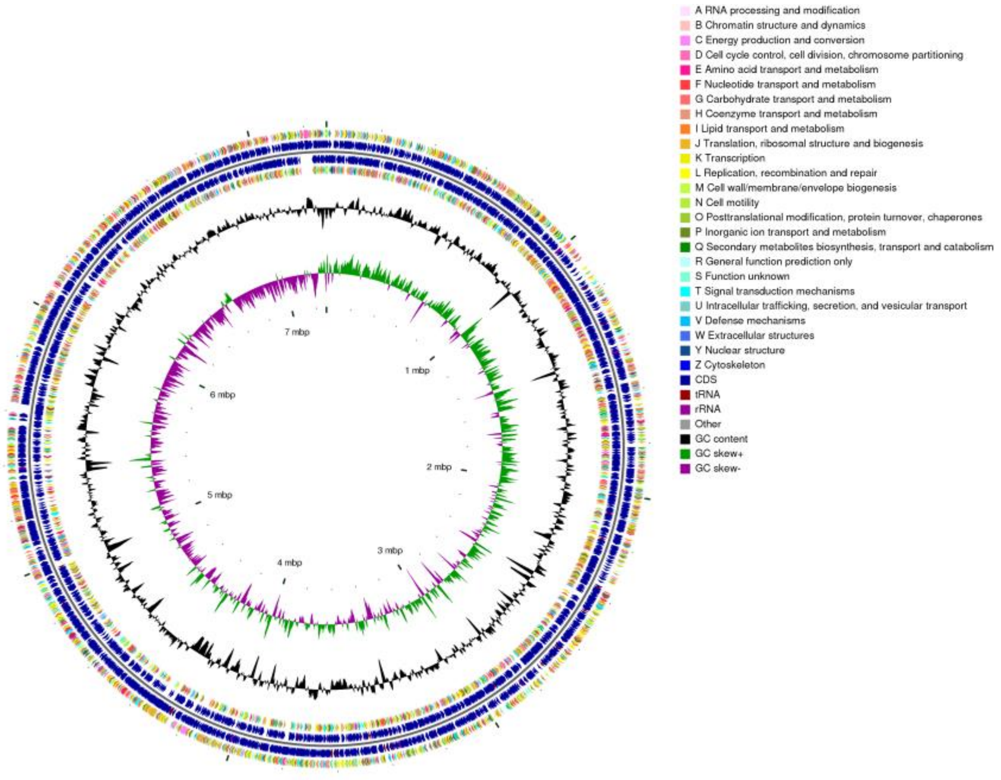

Figure S1. Circular genome map of actinomycete XH2.

Note: The first and fourth circles, moving from the outermost to the innermost, represent CDS on the positive and negative strands, with different colors indicating distinct COG functional classifications. The second and third circles depict CDS, tRNA, and rRNA on the positive and negative strands, respectively. The fifth circle illustrates the GC content, where outward or inward deviations show regions with GC content higher or lower than the genome-wide average, and the peak height reflects the magnitude of this deviation. The sixth circle represents the GC-skew, determined utilizing the formula (G–C)/(G+C), which aids in identifying the leading and lagging strands.

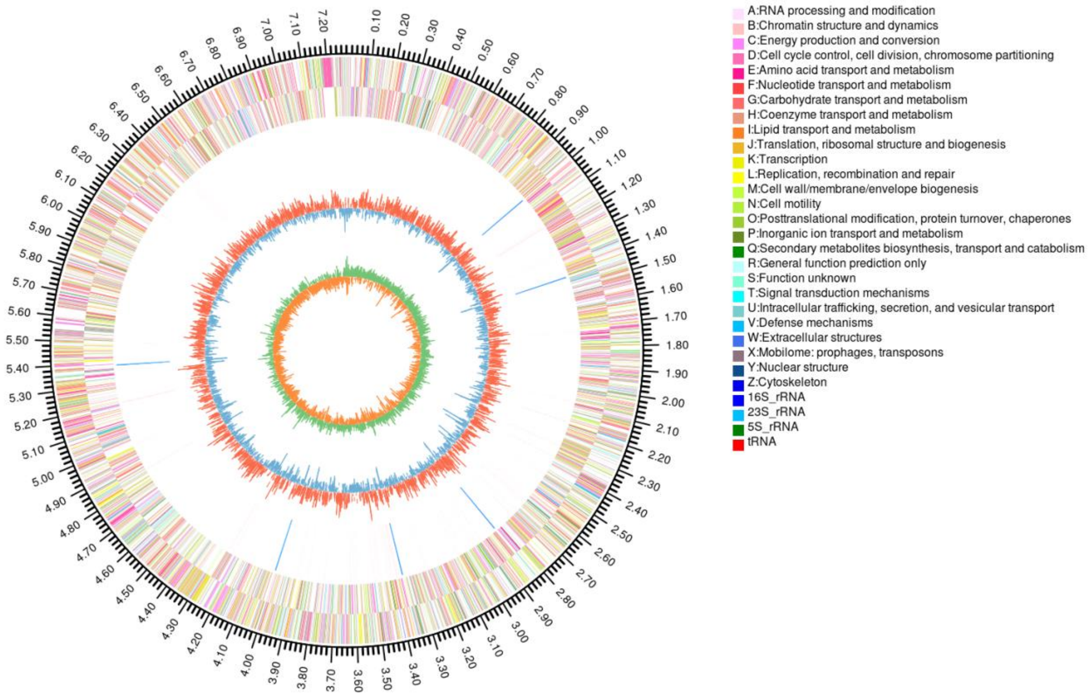

Figure S2. Circos plot of actinomycete XH2.

Note: The outermost ring of the circle plot indicates the genome size; the second and third rings represent CDSs on the positive and negative strands, with different colors indicating distinct COG functional classifications of the CDSs; The fourth circle shows rRNA and tRNA; the fifth circle displays GC content. The outward-facing red sections indicate GC content higher than the genome-wide average, with peak height reflecting the magnitude of deviation from the average. The inward-facing blue sections indicate GC content lower than the genome-wide average, with peak height reflecting the magnitude of deviation from the average. The innermost circle shows GC-Skew values, calculated as G-C/G+C, which aids in distinguishing the leading strand from the lagging strand.

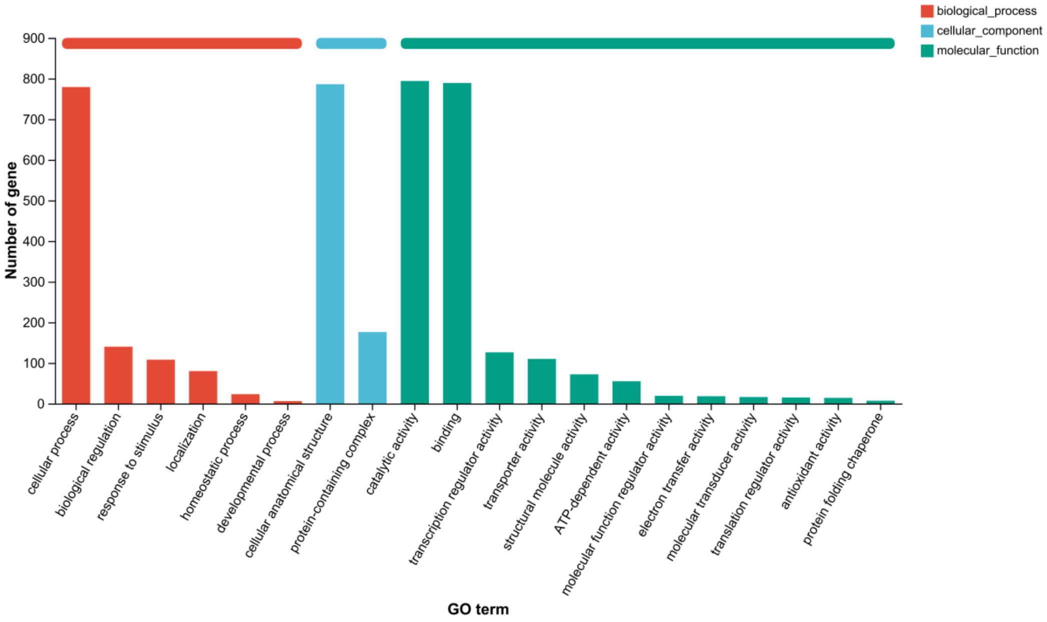

Figure S3. Statistical chart of GO annotation classification of transcriptomic sequencing results.

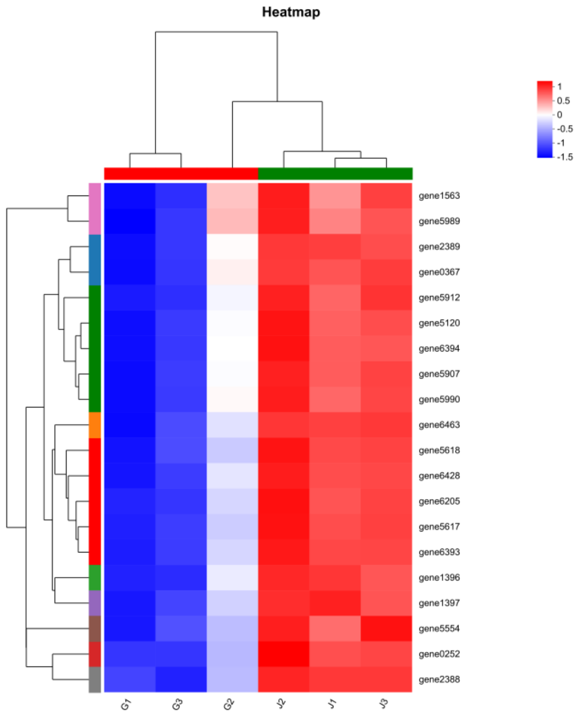

Figure S4. Heat map analysis of the top 20 genes in the transcriptome.
